# A multi-modal approach for the treatment of non-fluent/agrammatic variant of Primary Progressive Aphasia

**DOI:** 10.1093/braincomms/fcaf295

**Published:** 2025-09-03

**Authors:** Maria Cotelli, Ilaria Pagnoni, Elena Gobbi, Elena Campana, Sonia Bellini, Antonio Longobardi, Claudia Saraceno, Andrea Geviti, Valentina Cantoni, Antonella Alberici, Enrico Premi, Barbara Borroni, Roberta Ghidoni, Giuliano Binetti, Rosa Manenti

**Affiliations:** Neuropsychology Unit, IRCCS Istituto Centro San Giovanni di Dio Fatebenefratelli, Brescia 25125, Italy; Neuropsychology Unit, IRCCS Istituto Centro San Giovanni di Dio Fatebenefratelli, Brescia 25125, Italy; Neuropsychology Unit, IRCCS Istituto Centro San Giovanni di Dio Fatebenefratelli, Brescia 25125, Italy; Neuropsychology Unit, IRCCS Istituto Centro San Giovanni di Dio Fatebenefratelli, Brescia 25125, Italy; Molecular Markers Laboratory, IRCCS Istituto Centro San Giovanni di Dio Fatebenefratelli, Brescia 25125, Italy; Molecular Markers Laboratory, IRCCS Istituto Centro San Giovanni di Dio Fatebenefratelli, Brescia 25125, Italy; Molecular Markers Laboratory, IRCCS Istituto Centro San Giovanni di Dio Fatebenefratelli, Brescia 25125, Italy; Service of Statistics, IRCCS Istituto Centro San Giovanni di Dio Fatebenefratelli, Brescia 25125, Italy; Department of Clinical and Experimental Sciences, University of Brescia, Brescia 25123, Italy; Neurology Unit, ASST Spedali Civili di Brescia, Brescia 25123, Italy; Stroke Unit, Department of Neurological and Vision Sciences, ASST Spedali Civili, Brescia 25123, Italy; Molecular Markers Laboratory, IRCCS Istituto Centro San Giovanni di Dio Fatebenefratelli, Brescia 25125, Italy; Department of Clinical and Experimental Sciences, University of Brescia, Brescia 25123, Italy; Molecular Markers Laboratory, IRCCS Istituto Centro San Giovanni di Dio Fatebenefratelli, Brescia 25125, Italy; MAC Memory Clinic and Molecular Markers Laboratory, IRCCS Istituto Centro San Giovanni di Dio Fatebenefratelli, Brescia 25125, Italy; Neuropsychology Unit, IRCCS Istituto Centro San Giovanni di Dio Fatebenefratelli, Brescia 25125, Italy

**Keywords:** non-fluent/agrammatic variant of primary progressive aphasia, transcranial direct current stimulation, individualized language rehabilitation treatment

## Abstract

The non-fluent/agrammatic variant of primary progressive aphasia is a neurodegenerative disorder characterized by effortful language production and impaired comprehension of grammatically complex sentences. Recently, interest in non-pharmacological interventions has increased, particularly regarding techniques that allow for non-invasive brain stimulation, such as transcranial direct current stimulation. The main purpose of this study was to investigate whether the use of anodal transcranial direct current stimulation applied to the dorsolateral prefrontal cortex during individualized language training for 25 min a day at 5 days a week for 2 weeks would lead to significant oral naming improvements in patients with agrammatic variant of primary progressive aphasia. Specifically, we hypothesized that anodal transcranial direct current stimulation plus individualized language training may improve the oral naming of treated and untreated objects compared with both placebo transcranial direct current stimulation plus individualized language therapy and anodal transcranial direct current stimulation combined with computerized cognitive training. Forty-seven agrammatic variant of primary progressive aphasia patients were consecutively enrolled and randomized into one of three groups that received the following treatments: (i) anodal transcranial direct current stimulation over the left dorsolateral prefrontal cortex during individualized language rehabilitation treatment; (ii) placebo transcranial direct current stimulation during individualized language rehabilitation treatment; or (iii) anodal transcranial direct current stimulation with computerized cognitive training. Clinical, neuropsychological and language assessments were recorded at baseline (T0), post-treatment (T1, 2 weeks) and at 12 weeks from T0 (T2). Magnetic resonance imaging data, functional magnetic resonance imaging data and blood samples were collected at T0 and T1. All of the groups demonstrated improvements in oral object naming at T1, with maintenance effects being observed at T2. At T1, the enhancement in the oral naming of treated and untreated objects was significantly greater in patients who underwent anodal transcranial direct current stimulation during individualized language rehabilitation treatment. There were no significant changes observed across the groups regarding the magnetic resonance imaging, functional magnetic resonance imaging or blood biochemical marker data. Our results support the beneficial effects of individualized language rehabilitation treatment in combination with anodal transcranial direct current stimulation in agrammatic variant of primary progressive aphasia patients.

## Teaser

Cotelli *et al*. investigated the effects of combined anodal transcranial direct current stimulation (tDCS) and individualized language rehabilitation on oral naming in patients with non-fluent/agrammatic primary progressive aphasia. Results showed that combined treatment improved oral naming abilities, with effects maintained over time, compared with language rehabilitation or tDCS alone.

## Graphical Abstract

**Graphical Abstract:**
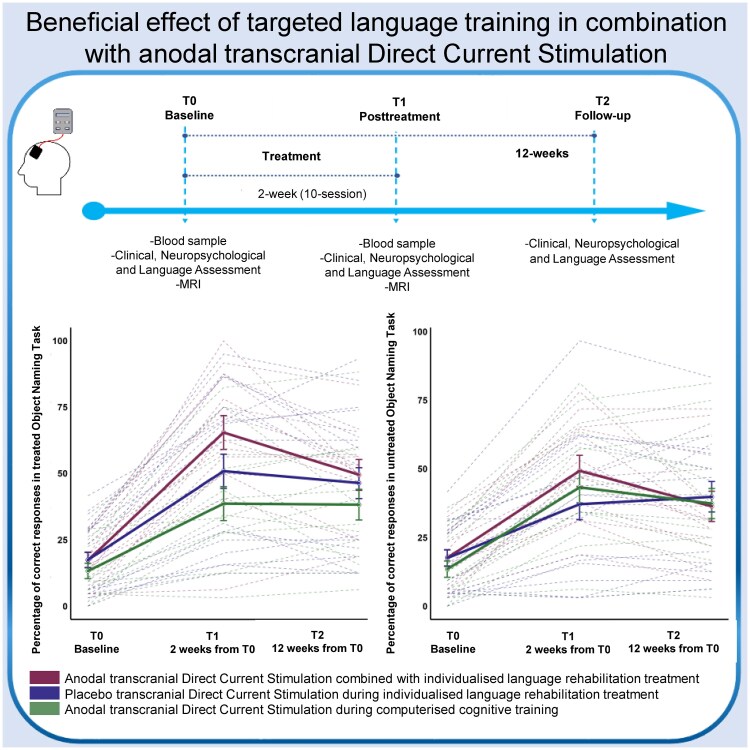

## Introduction

The investigation of alterations affecting different levels of linguistic organization, as well as communication skills and cognitive functioning observable in the clinical course of primary progressive aphasia (PPA), has revealed the neurobiology of language with a novel and complementary perspective to the classical perspective, which has focused on aphasia caused by focal lesions.^1^ Furthermore, the possibility of investigating the neurological basis of language disorders via advanced neuroimaging techniques, which has allowed for the investigation of the structure and functioning of the brain *in vivo* via anatomical and functional connectivity studies, has significantly contributed to the growth of interest in this field of language pathology.

PPA refers to a collection of anatomically and pathologically heterogeneous neurodegenerative diseases^2-12^ characterized by a gradual, insidious and progressive loss of language skills.^13-16^ To establish a diagnosis of PPA, the following three criteria must be met: (i) the patient must present with a language disorder evident in language production or comprehension not due to motor or perceptual deficits; (ii) the language disorder should represent the main deficit in the early stages of the disease and also represent the major obstacle in daily living activities; and (iii) the disease must be of a neurodegenerative nature and consequently demonstrate a progressive trend.^16-18^

Currently, based on the most frequently observed symptoms, Gorno-Tempini *et al*.^5^ classified PPA into three main variants, including the non-fluent/agrammatic variant (avPPA), semantic variant (svPPA) and logopenic/phonological variant (l/phvPPA), with the classification of these variants based on neurolinguistic, neuropsychological, neuroanatomical and neuropathological aspects.

The non-fluent/agrammatic variant of PPA (avPPA) is characterized by slow, effortful and hesitant language production, including grammatical errors and omissions,^4,6,16,19,20^ as well as greater difficulties in naming actions than objects.^21,22^ The core diagnostic features of this variant include agrammatism and speech sound errors with distortions (known as apraxia of speech or AOS), with the presence of at least one being required for diagnosis.^5^ Moreover, comprehension is spared, except for grammatically complex sentences.^5,23^ The avPPA variant is associated with atrophy in the left inferior frontal gyrus, supplementary motor areas and insula.^5,24-26^ In contrast, the semantic variant of PPA (svPPA) is characterized by fluent speech and semantic memory difficulties, thereby leading to issues in naming tasks and understanding single words^27^; additionally, this variant presents with atrophy of the anterior temporal lobe.^24,28,29^ The logopenic/phonological variant (l/phvPPA) is characterized by slow speech, with frequent pauses and more evident anomies being observed for the retrieval of long words. Additionally, l/phvPPA is associated with difficulties in repetition tasks and syntactic comprehension tests; moreover, it presents with atrophy of the left posterior temporal cortex and left parietal lobule.^3,24,26,30-32^ Furthermore, the three different phenotypes have been associated with distinctive underlying autoptic determinants, with Tau depositions contributing to avPPA, transactive response DNA binding protein 43 (TDP-43) proteinopathy typically present in svPPA, and Alzheimer’s disease pathology typically detected in l/phvPPA.^33^

Word retrieval difficulties are commonly observed in PPAs, and impaired naming ability is often one of the first symptoms experienced by these individuals.^34-36^ Several interventions for anomia in PPA patients have been developed,^37-39^ when considering that the nature of the naming deficit may differ. In contrast to svPPA and l/phvPPA, in which naming errors reflect the loss of semantic representations^40,41^ and a deficit in lexico-phonological abilities,^31^ anomia arises in avPPA from a post-semantic deficit at the level of the phonological or orthographic output lexicon.^42,43^

The aim of the impairment-based/restitutive approach tailored to the language deficits of the PPA is to minimize the functional impacts of communication difficulties on activities of daily living and to rehabilitate specific language deficits.^39,44-56^ Despite the neurodegenerative nature of the disease and its heterogeneous variants,^57^ research suggests that treatments targeting word retrieval, including semantic, phonological and orthographic approaches, yield considerable benefits in naming performance among PPA patients, along with a generalized effect on functional communication.^46,58-61^

In the last few years, interest in non-invasive human brain stimulation techniques, such as repetitive transcranial magnetic stimulation (rTMS) and transcranial direct current stimulation (tDCS), has increased. There is evidence that both techniques can modify cortical plasticity by increasing excitability in specific cortical neuron networks, thus resulting in improved cognitive abilities.^62-72^ The brain changes induced by these brain stimulation methods have been demonstrated to persist beyond the stimulation period, which implies that it is possible to manipulate brain excitability and possibly facilitate neural plasticity phenomena.^73^ Recent studies have supported the benefits of the use of targeted language training in combination with brain stimulation in PPA^39,72,74-86^; however, only a few studies have investigated the neural correlates associated with improvements from cognitive and tDCS rehabilitation protocols with the aim of identifying the variables associated with successful recovery from aphasia.^69,74,87,88^ Brain-derived neurotrophic factor (BDNF) and neurogranin are known markers of synaptic plasticity (which is defined as the ability of the brain to adapt and reorganize itself) and are particularly important for learning and memory processes. BDNF is vital for synaptic plasticity; however, it is also involved in neuronal differentiation, maturation, survival and protection.^89,90^ Even neurogranin has been implicated in the modulation of synaptic strength and plasticity (which is mediated by calmodulin and calcium signalling pathways) and in the maturation and differentiation of neurons.^91,92^ Both altered BDNF and neurogranin levels have been observed to be associated with various neurodegenerative and neuropsychiatric diseases, including Alzheimer’s disease, Parkinson’s disease, major depressive disorder, schizophrenia and anxiety-related disorders.^93,94^ Given their ability to modulate synaptic transmission and plasticity, BDNF and neurogranin can be used to monitor the effectiveness of individualized language rehabilitation treatment in combination with anodal tDCS. It has been demonstrated that behavioural interventions (such as cognitive behavioural therapy and physical exercise) can influence BDNF levels in psychiatric and neurodegenerative patients.^95,96^ Moreover, structural and functional neuroimaging techniques have been previously used to explore the brain-behavioural relationship in neurodegenerative diseases,^97-101^ as well as its role as a potential surrogate marker of efficacy for neuromodulation trials.^76,82,102,103^ Therefore, in the present study, we implemented the following neuroimaging techniques to explore treatment-induced neuroimaging markers in avPPA patients: (i) structural analysis to explore the changes in grey matter density at the whole-brain level (using voxel-based morphometry, VBM), as well as a hypothesis-driven approach (region of interest (ROI) for the left dorsolateral prefrontal cortex, DLPFC); and (ii) functional connectivity analysis focused on large-scale networks (including salience, language and default mode networks (DMN)), as well as subdivisions of the central executive network.

The primary objective of the present study was to evaluate whether the application of anodal tDCS (the anode located over the left DLPFC and the cathode located over the right supraorbital region) during individualized language rehabilitation treatment focused on object naming skills could improve oral naming ability in patients with avPPA. Specifically, we hypothesized that this protocol would facilitate performance in the oral naming of treated and untreated object tasks. To address this question, we compared the effects of anodal tDCS (atDCS) or placebo tDCS (ptDCS) combined with individualized language rehabilitation treatment (atDCS-Lang or ptDCS-Lang) with those of atDCS combined with computerized cognitive training (atDCS-Cog) on performance in oral naming tasks. Moreover, we directly compared the three training groups to observe additional gains from combined treatment (atDCS-Lang). In particular, this study was designed to: (i) evaluate the efficacy of individualized language rehabilitation treatment combined with anodal tDCS for oral naming compared with placebo tDCS stimulation during individualized language rehabilitation treatment and anodal tDCS combined with computerized cognitive training; (ii) determine the long-lasting beneficial effects of these interventions; and (iii) map neuroimaging and blood marker changes associated with improvements in language performance. To achieve this goal, we evaluated patients with avPPA at baseline (T0), immediately after the intervention (T1, 2 weeks) and at 12 weeks from T0 (T2) using neuropsychological assessments, whereas neuroimaging (MRI and functional fMRI) and biochemical investigations (plasma BDNF and neurogranin) were conducted at T0 and T1.

## Materials and methods

### Participants

This study included patients with a non-fluent/agrammatic variant of PPA (avPPA) who met the diagnostic criteria for clinical and imaging-supported avPPA.^5^

The inclusion criteria for the participants were as follows: (i) native Italian speakers; (ii) had normal or corrected-to-normal vision and hearing and were medically stable; (iii) had a CDR plus NACC FTLD (global clinical dementia rating plus National Alzheimer’s Coordinating Center Frontotemporal Lobar Degeneration) score between 0.5 and 2^104,105^; (iv) lacked non-degenerative neurologic disorders; and (v) agreed to participate in the study by signing informed consent forms.

The exclusion criteria included the presence of any psychiatric or medical illness that could interfere in completing the assessments, a history of significant drug or alcohol abuse, a history of brain surgery and the presence of any medical condition that represents a contraindication to tDCS (such as a history of seizures or metal located in the head). The diagnostic evaluation included neuropsychological tests, language evaluations, neurologic examinations and neuroimaging (including MRI or PET).

In addition, patients were included in the study after formal language assessments were performed via the Screening for Aphasia in NeuroDegeneration battery (SAND), which revealed a prevalence of agrammatism rather than apraxia of speech.^106,107^

Accordingly, 83% of the patients demonstrated morphological errors in the auditory sentence comprehension test, 66% of the patients demonstrated grammatical/syntactic errors in the writing subtest, 34% of the patients demonstrated grammatical/syntactic errors in the picture description subtest and 11% of the patients demonstrated morphological errors in the sentence repetition subtest. Apraxia of speech was not a prevalent feature of our avPPA sample, as demonstrated by the low percentage of patients reporting of articulatory errors/distortions in the picture description subtest (17%), sentence repetition subtest (4%) and non-word repetition subtest (2%). Moreover, articulatory distortions were not observed in the other oral production tasks (such as picture naming, reading and word repetition subtests).

### Study design

This multi-centric study was a double-blinded, randomized, placebo-controlled pilot study involving 47 patients with avPPA.^5^ Participants were recruited from the IRCCS Istituto Centro San Giovanni di Dio, Brescia, Italy, and the ASST Spedali Civili, Brescia, Italy, between February 2020 and March 2023.

The protocol of the present study was prepared as outlined in the ‘Standard Protocol Items: Recommendations for Interventional Trials’ (SPIRIT) guidelines^108^ (Table 1).

**Table 1:** Schedule of enrolment, intervention and assessment in the study (SPIRIT)

Time point	Enrolment	T0 baseline	T1 2 weeks from T0	T2 12 weeks from T0
Enrolment				
Eligibility screening	X			
Informed consent	X			
Allocation		X		
Interventions				
Anodal tDCS during individualized language training		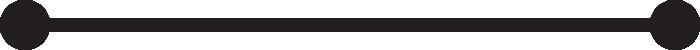		
Placebo tDCS during individualized language training		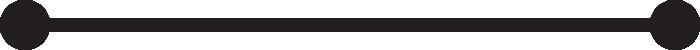		
Anodal tDCS during computerized cognitive training		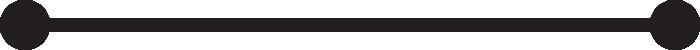		
Assessments				
Baseline measures				
EHI		X		
CRI-q		X		
CDR plus NACC FTLD		X		
Primary outcome measures				
IPNP—treated object naming task		X	X	X
IPNP—untreated object naming task		X	X	X
IPNP—action naming task		X	X	X
Secondary outcome measures				
BDI		X	X	X
FBI		X	X	X
SAQoL-39		X	X	X
Lincoln speech questionnaire		X	X	X
ASRS		X	X	X
Story recall		X	X	X
ROCF—copy and recall		X	X	X
TMT		X	X	X
Verbal fluency (semantic and phonemic)		X	X	X
AAT—oral naming subtest		X	X	X
SAND		X	X	X
Surrogate outcome markers				
BDNF levels (pg/ml)		X	X	
Neurogranin levels (pg/ml)		X	X	
Resting-state functional-MRI		X	X	
Structural-MRI		X	X	
Other measures				
tDCS—adverse events questionnaire		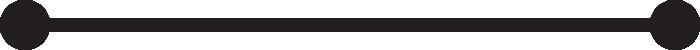		
tDCS—sensations questionnaire		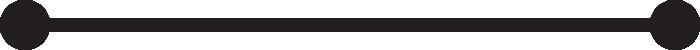		
Blinding efficacy question			X	

AAT, Aachener Aphasia test; ASRS, Aphasia severity rating scale; BDI, Beck Depression Inventory; BDNF, brain-derived neurotrophic factor; CDR plus NACC FTLD, global clinical dementia rating plus National Alzheimer’s Coordinating Center Frontotemporal Lobar Degeneration; CRI-q, cognitive reserve index-questionnaire; EHI, Edinburgh handedness inventory; FBI, frontal behavioural inventory; IPNP, international picture naming project; pg/ml, picograms per millilitre; ROCF, Rey–Osterrieth complex figure; SAND, screening for aphasia in neurodegeneration; SAQoL-39, stroke and aphasia quality of life scale-39; tDCS, transcranial direct current stimulation; T0, baseline assessment; T1, post-treatment assessment; T2, 12 weeks from T0 assessment; TMT, trail making test.

This study was conducted in accordance with the Declaration of Helsinki and approved by the Ethics Committees of the IRCCS Istituto Centro San Giovanni di Dio, Fatebenefratelli, Brescia, Italy (protocol number: 42-2019) and of the ASST Spedali Civili, Brescia (protocol number: 3783).

After receiving information about the study and only after a safety screening regarding the possible risks of tDCS, all of the participants freely chose to participate in the study by signing a written informed consent form. The trial was registered at clinicaltrials.gov (NCT number: 04187391).

Eligible patients who met all of the inclusion criteria were randomized (1:1:1) using an adaptive randomization procedure considering age and oral naming difficulties (according to the raw score on the naming subtest from the Aachener Aphasia test, or AAT^109^). Stratified randomization was implemented by creating separate blocks for each combination of covariates. The participants were assigned to the appropriate covariate block by a researcher who was blinded to the study aims. Allocation details were provided on cards placed into sequentially numbered, opaque and sealed envelopes. Investigators, clinicians and outcome assessors, as well as the statistician who performed the statistical analysis, were blinded to the group allocation.

All of the subjects were randomized into one of the following three treatment groups:

- Anodal tDCS over the left DLPFC combined with individualized language rehabilitation treatment (atDCS-Lang).
- Placebo tDCS over the left DLPFC during individualized language rehabilitation treatment (ptDCS-Lang).
- Anodal tDCS over the left DLPFC during computerized cognitive training (atDCS-Cog).

**Figure 1:**
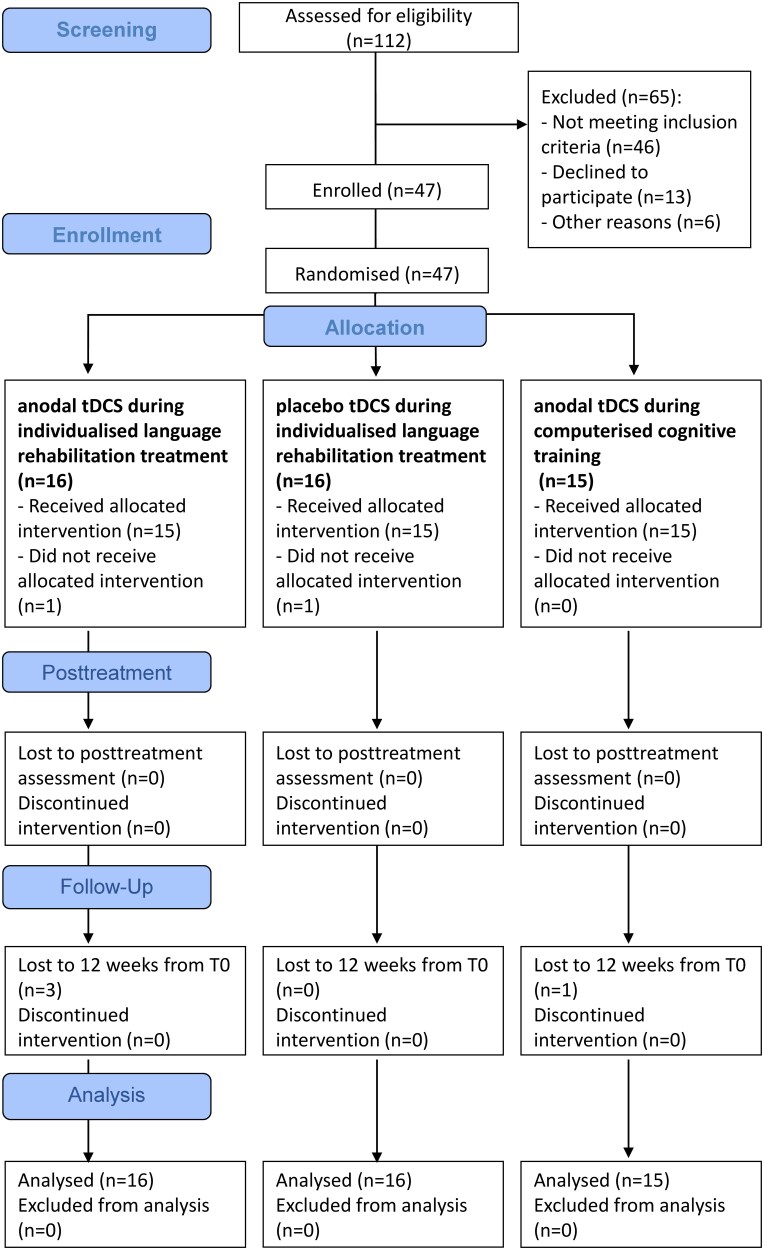
**Consort flow diagram.** Flow chart showing study subject enrolment and sample processing. tDCS, transcranial direct current stimulation; T0, baseline assessment.

**Figure 2:**
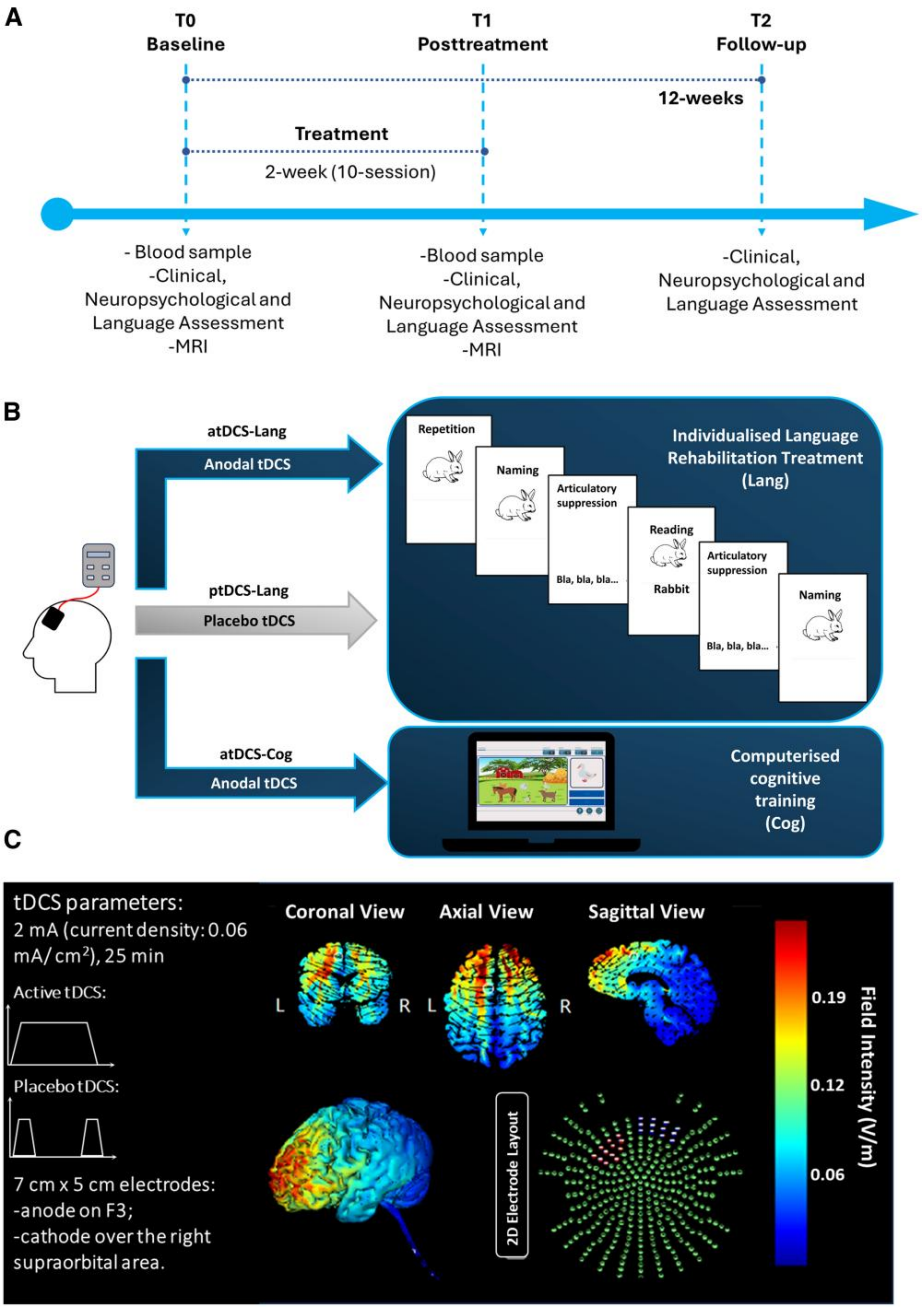
**Study design.** (**A**) Timeline for the experimental protocol. (**B**) Treatment protocols: anodal tDCS over left DLPFC combined with an individualized language rehabilitation treatment (atDCS-Lang); placebo tDCS over left DLPFC during individualized language rehabilitation treatment (ptDCS-Lang); anodal tDCS over left DLPFC during computerized cognitive training (atDCS-Cog). (**C**) Current flow model of tDCS montage (anode over F3 and cathode over the right supraorbital area), using two 7 × 5 cm sponge pads represented in axial, sagittal and coronal views from the Male 1 model in the Soterix HD targets software (Soterix medical). Arrows represent the direction of current flow. cm, centimetre; DLPFC, dorsolateral prefrontal cortex; L, left; mA, milliampere; min, minutes; R, right; tDCS, transcranial direct current stimulation; T0, baseline assessment; T1, post-treatment assessment; T2, 12 weeks from T0 assessment; V/m, volt per metre.

All of the participants underwent a clinical, neuropsychological and language evaluation at baseline (T0), at post-treatment (T1, 2 weeks) and at 12 weeks from T0 (T2). Additionally, MRI, fMRI and blood sample data were collected at T0 and T1 (before and after treatment). See Figs. 1 and 2A for more details. All of the procedures were conducted at the IRCCS Istituto Centro San Giovanni di Dio Fatebenefratelli, Brescia, except for the MRI scans, which were acquired at the ASST Spedali Civili Brescia.

The sample size was determined using G*Power 3 software. For this study, the calculation was based on prior research^75^ and was calculated for the oral naming of the treated subjects (the primary outcome variable). An eta squared value of 0.25 for the interaction between time and the experimental group was used for the effect size calculation. To achieve 90% power with an alpha level of 0.05, a minimum of 12 subjects was required. This number was then increased to 15 subjects per group to accommodate an expected dropout rate of 20%.

### Clinical, neuropsychological and language assessments

Data regarding demographic characteristics, the complete medical history, current drug therapy and results of the complete neurological examination were collected during the baseline evaluation (T0). Similarly, screening tools for handedness (such as the Edinburgh Handedness Inventory, or EHI),^110^ the screening tDCS checklist, the CDR plus NACC FTLD scale^104,105^ and the cognitive reserve index-questionnaire (CRI-q)^111^ were administered at the baseline assessment (T0). The CDR plus NACC FTLD scale was administered to measure the severity of dementia and progression to FTLD^105^ and the CRI-q^111^ was implemented to assess the level of cognitive reserve via three subscales that investigate different life domains (CRI-education, CRI-work activity and CRI-leisure). An index was calculated for each of these subscales, and the average of these indices yields a final total score (CRI-total score), which can be classified into five levels: low (<70), medium–low (70–84), medium (85–114), medium–high (115–130) and high (more than 130).^111^

The evaluation of communication and functional abilities was conducted using the Stroke and Aphasia Quality of Life Scale (SAQOL-39).^112^ The SAQOL-39 is based on four subdomains: physical, psychosocial/mood, communication and energy. The Lincoln speech questionnaire^113^ and the Aphasia severity rating scale (ASRS)^114^ were also used. Depression was assessed with the Beck Depression Inventory (BDI),^115^ whereas personality and behavioural changes were recorded using the frontal behavioural inventory (FBI).^116^

The neuropsychological assessment included the story recall^117^ and the Rey–Osterrieth complex figure test-recall^118^ for episodic memory; the Rey–Osterrieth complex figure test-copy^118^ for visuoconstructional abilities; and the trail making test (TMT) Part A and Part B^119^ for attention functions. All of the tests were administered and scored according to standard procedures.^120^

Linguistic abilities were evaluated via phonemic and semantic verbal fluency^121^ for language production, including the naming subtest from AAT,^109^ an action naming task (the International Picture Naming Project, or IPNP^122^) and the SAND battery.^106,107^ The SAND is a screening battery for language assessment and includes nine tests: picture naming, auditory sentence comprehension, single-word comprehension, word and non-word repetition, sentence repetition, reading, writing, semantic association and picture description.

The oral tasks (including the subtest from AAT, the action naming task from the IPNP and the oral tasks of the SAND) were audio-recorded using the Audacity software (v.2.3; https://www.audacityteam.org/) and subsequently analysed offline for accuracy.

The same trained neuropsychologist performed all evaluations (T0, T1 and T2), remaining blinded to treatment allocation but not to the timing of each visit.

### Procedure to select individualized ‘treated’ and ‘untreated’ object item lists for the oral object naming task

At baseline (T0), to select individualized ‘treated’ and ‘untreated’ object item lists for the oral object naming task assessment, all of the patients underwent two sessions of oral object naming to select two individualized sets of object stimuli for use in the oral object naming task at all of the assessment time points. Pictures for the oral naming task included 300 black and white drawings of objects taken from the UCSD Centre for Research in Language-IPNP corpus.^122^ The IPNP database provides standards and lexical information (including data regarding frequency and age of acquisition, among other information) for picture naming in seven languages. The pictures have been tested and standardized in both healthy and patient populations.^122^

Stimuli for the oral naming task were presented twice (on two consecutive days) on a computer screen using Presentation software (https://www.neurobs.com/, v.22.0); each picture was presented for a maximum of 10 s, during which the participants were asked to name the object, and oral responses were recorded.

Oral responses were recorded using the Audacity software (v.2.3; https://www.audacityteam.org/) and subsequently analysed offline for accuracy.

Distortions and substitutions of only one phoneme were scored as corrected. Additionally, due to the fact that the therapy aimed to focus selectively on nouns with lexical production difficulties and not nouns related to semantic difficulties, all of the pictures that were incorrectly named in at least one of the two oral naming sessions were included in a subsequent comprehension task to ensure that the participants understood all of the item meanings. In the comprehension task, the participants were asked whether a picture presented for a maximum of 10 s corresponded or did not correspond to the spoken word, after which they had to answer dichotomously (either yes or no). The participant was questioned about the name of the picture during three consecutive trials, including the picture’s correct name, a semantic distractor and an unrelated distractor (e.g. for the picture of a bottle, the questions were ‘Is it a bottle?’, ‘Is it a glass?’ or ‘Is it a calendar?’, respectively). Only items for which no errors were recorded were selected. The individually selected pictures were further categorized into two balanced sets: the ‘treated object’ list (32 items) and the ‘untreated object’ list (32 items). The two lists were balanced using a *t*-test (all *P-*values were >0.05) for a number of variables related to the participant’s performance, such as the percentage of correct oral picture naming across assessment sessions, number of syllables and letters of the words, target word frequency and semantic category (i.e. living or non-living).^123^ The procedure that was applied to select the two lists produced a personalized set of items for each participant, which ensured both within-subject and across-subject validity of the design. For the patients who underwent the language treatment, the ‘treated object’ list included the object pictures that were selected for the individualized language rehabilitation treatment, and the ‘untreated object’ list included the object pictures that were used as control stimuli. For the patients assigned to the computerized cognitive training group, neither list of stimuli was treated via language treatment.

### Interventions

The participants received treatment sessions of 25 min for five consecutive days a week for 2 weeks, according to their group allocation. The treatment approaches included in this protocol are detailed in the following paragraphs.

#### tDCS protocol

All of the patients received two weeks of tDCS stimulation over the left DLPFC (active/anodal or placebo stimulation, depending on the assigned group) in combination with a specific treatment (individualized language rehabilitation treatment or computerized cognitive training; Fig. 2B and C).

With respect to oral naming, we targeted the left DLPFC during stimulation, which aligned with previous studies that have used brain stimulation to demonstrate this region’s involvement in naming abilities in young adults, healthy older adults and clinical populations.^21,64-66,124-129^ Furthermore, this area was selected due to the predominantly left-sided DLPFC atrophy observed in individuals with avPPA, with the goal of enhancing its functional activity.^4,130^

We delivered the stimulation using a battery-driven constant-current stimulator (BrainStim, EMS, Bologna, Italy) through a pair of saline-soaked sponge electrodes (35 cm^2^ each). The anode electrode (7 × 5 cm) was placed on the left DLPFC (F3) at positions of 8 cm frontally and 6 cm laterally relative to the scalp vertex, and the reference electrode was fixed over the right supraorbital region, according to the 10–20 EEG international system.^131^

A constant current of 2 mA was applied for 25 min (current density: 0.057 mA/cm^2^) starting at the beginning of the training (Lang or Cog), with a ramping period of 10 s being utilized at both the beginning and the end of the stimulation (Fig. 2B and C).^132-134^ The current density was maintained below the safety limits.^132-134^ Fig. 2C displays a graphical representation of the computerized modelling of tDCS-induced current flow (Soterix Medical; https://soterixmedical.com). For the sham stimulation (i.e. the placebo), the current was turned off 10 s after stimulation began (plus the duration of the fade-in) and was activated during the final 10 s of the stimulation period, including the fade-out phase, which also lasted 10 s. The patients experienced itching sensations below the electrodes at both the beginning and end of the stimulation, which indicated that this condition was indistinguishable from the experimental stimulation.^135^

At the end of each tDCS session, participants completed questionnaires to assess any adverse events and sensations induced by tDCS.^136^ After completing the last treatment session, they were also asked to indicate whether they believed they had received anodal or sham tDCS stimulation (with responses recorded in a dichotomous way).

#### Individualized language rehabilitation treatment (Lang)

The subjects who were assigned to the atDCS-language rehabilitation treatment (Lang) and ptDCS-Lang groups were seated in front of a computer screen in a quiet room while the individualized language rehabilitation treatment protocol focusing on oral object naming skills was implemented during anodal or placebo tDCS (depending on the assigned group). The individualized language rehabilitation treatment included several steps to encourage the strategic recruitment of spared phonological and orthographic knowledge, in order to facilitate word retrieval and to elicit the production of the target noun.^72,137,138^ For this purpose, the training was designed to include elements of the treatment most widely used in clinical practice, such as lexical retrieval treatment^46^ and phonological and orthographic treatment.^139-141^ Moreover, the training included an articulatory suppression task, in which patients were asked to repeat some irrelevant speech sound that could interfere with the ability to rehearse the phonological information.^142^ At each step, in the case of difficulties or errors, feedback was provided to the patients and the patients were corrected until they provided the correct response.

The structure of the Lang protocol was as follows (Fig. 2B):

Step 1—oral repetition: the therapist spoke the target word, and the participant was asked to repeat it three times.

Step 2—articulatory suppression task: this step involved interference with articulatory codes caused by the uttering of an irrelevant speech sound (i.e. bla, bla, bla).

Step 3—oral picture naming: the target picture was presented on the computer screen, and the participant was asked to retrieve its correct name.

Step 4—oral reading: the target written word was presented on the computer screen, and the participant was asked to read it.

Step 5—articulatory suppression task: this step involved interference with articulatory codes caused by the uttering of an irrelevant speech sound (i.e. bla, bla, bla).

Step 6—oral picture naming: the target picture was presented on the computer screen, and the participant was asked to retrieve its correct name.

#### Computerized cognitive training (Cog)

The participants who were assigned to the computerized cognitive training (Cog) group were seated in a quiet room while the cognitive virtual reality rehabilitation system (VRRS) treatment (http://khymeia.com/) was delivered during anodal tDCS (Fig. 2B). Ten exercises (designed to enhance visuospatial and visual-perceptual abilities) were selected (listed in Supplementary Table 1). In each treatment session, a participant worked with five exercises for 5 min each; moreover, task difficulty did not adaptively progress. Computerized cognitive training (Cog) was provided with a touchscreen tablet (VRRS-Tablet). The subject was asked to continue performing each task until the end of the set time period.

### Outcome measures

#### Primary outcome measures

The primary outcome measure involved the changes from baseline (T0) to post-treatment (T1) and follow-up assessment (T2) in oral object and action naming task scores, as assessed via the IPNP task.^122^

To evaluate the accuracy of these picture naming tasks (object and action naming), the participants were seated in a dimly lit room facing a computer monitor that was located 60 cm away. The stimuli were presented using Presentation software (version 22.0, www.neurobs.com) on a personal computer with a 15-inch screen. Verbal responses were recorded and digitized via Audacity software (v.2.3; https://www.audacityteam.org/). The oral action naming test was included to assess the generalizability to the naming of untrained action items.^122^

#### Secondary outcome measures

The secondary outcome measures included the changes from baseline (T0) to post-treatment (T1) and follow-up assessment (T2) in the standardized clinical, communication and functional ability questionnaires, as well as neuropsychological tests for each cognitive domain and specific language task.

#### Surrogate outcome markers

The surrogate outcome measures included changes from baseline (T0) to post-treatment (T1) in blood biochemical markers and structural and functional MRI analyses.

1. Blood biochemical markers: plasma levels of BDNF and neurogranin were measured in duplicate in samples with available blood samples at both T0 and T1 (*n* = 45). BDNF plasma concentrations were measured with a Luminex Discovery Assay Kit (BioTechne R&D Systems®, Minneapolis, MN) following the manufacturer’s protocol. The neurogranin plasma concentration was evaluated with a Neurogranin (NRGN) Human ProcartaPlex™ Simplex Kit Panel combined with a ProcartaPlex Human Basic Kit (Invitrogen, Thermo Fisher Scientific Inc., Waltham, MA) following the manufacturer’s protocol. The plasma samples were diluted 1:2 and 1:10 for BDNF and neurogranin dosages, respectively. Analyses were performed on a Bio-Plex® 200 System (Bio-Rad, Hercules, CA) equipped with Bio-Plex® Manager™ Software 6.0 (Bio-Rad, Hercules, CA).
2. Structural MRI analysis: brain structural images (three-dimensional T1-weighted magnetization-prepared rapid acquisition with gradient echo (MPRAGE) MRI) were obtained using Siemens Skyra 3T (repetition time [TR] = 2000ms, echo time [TE] = 2.92 ms, pixel size = 1.1016 × 1.1016 mm^2^, pixel spacing = 1.1015625/1.1015625, acquisition matrix: 0 × 256 × 256 × 0). The original DICOM scans were converted to the Neuroimaging Informatics Technology Initiative (NIfTI) format using MRIcroGL software (www.nitrc.org/projects/mricrogl). The T1-weighted images were processed and analysed using the VBM pipeline in the Computational Anatomy Toolbox (CAT12 v.1742) (www.neuro.uni-jena.de/cat) for Statistical Parametric Mapping (SPM12, v.7219) (www.fil.ion.ucl.ac.uk/spm/software/spm12) in MATLAB R2023b (MathWorks, Inc., Natick, Massachusetts, USA). Furthermore, when considering the aim of the study (the longitudinal effect of the multimodal approach on PPA), a specific pre-processing pipeline for longitudinal data was adopted (optimized for detecting small changes, such as brain plasticity or training effects after a few weeks or even shorter periods of time). This VBM pipeline (which is currently implemented in the CAT12 toolbox) involves tissue segmentation, spatial normalization to a standard Montreal National Institute (MNI) template, modulation and smoothing,^143^ thus providing robust and accurate performance in calculating grey matter volume (GMV) compared with other VBM pipelines.^144^ The normalized and modulated grey matter images were subsequently smoothed with a Gaussian kernel (8 × 8 × 8 mm full width at a half-maximum) (see the supplementary materials for more details). Moreover, a ROI was defined for the left DLPFC (as the target region for tDCS) via the WFU Pickatlas toolbox (https://www.nitrc.org/projects/wfu_pickatlas, v.3.0.5) implemented in SPM12, with consideration of Broadmann areas 9 and 46.
  A flexible factorial design for whole-brain voxel-wise analysis was implemented in SPM12 to test the efficacy of the interventions, with consideration of the normalized and modulated grey matter images of the three groups of subjects (atDCS-Lang, ptDCS-Lang and atDCS-Cog) across two time points (T0 and T1). A comprehensive range of nuisance variables, such as age, sex, disease stage (CDR plus NACC FTLD) and total intracranial volume (TIV), was considered. A statistical threshold of *P* < 0.001 (uncorrected for multiple comparisons) was established, with a minimum cluster size of 100 voxels. For ROI-based analysis, generalized linear mixed models (GLMMs) were used to assess left DLPFC GMV variations between the groups over time. Moreover, partial correlation analysis (considering age, sex, disease stage (CDR plus NACC FTLD) and TIV as nuisance variables) was used to assess the relationship between the variation in left DLPFC volume (delta T1-T0) and naming improvements (delta T1-T0). A statistical threshold of *P* < 0.05 was used for this analysis.
3. Functional connectivity MRI analysis: T2-weighted echo planar imaging (EPI) sequences sensitized to blood oxygenation level-dependent contrast for resting state functional MRI (rs-fMRI) were used in the present study (3T Siemens Skyra: 200 time points; total acquisition time: 6 min and 20 s; TR = 2500 ms; TE = 30 ms; slice thickness = 3.5 mm; acquisition matrix = 64 × 0 × 0 × 64; flip angle = 80°; pixel size = 3 × 3 mm^2^). During scanning, the patients were asked to keep their eyes closed, not to think of anything in particular, and not to fall asleep. The functional data were pre-processed using the toolbox for data processing and analysis for brain imaging (DPABI, http://rfmri.org/dpabi)^145^ via SPM12 (https://www.fil.ion.ucl.ac.uk/spm/) software. For each subject, the first five volumes of the fMRI series were discharged to account for magnetization equilibration. The remaining 195 volumes underwent slice-timing correction and were realigned to the first volume. Any patient who had a maximum displacement in any direction larger than 2.5 mm or a maximum rotation (x, y and z) larger than 2.5° was excluded. We considered frame-wise displacement (FD) as a relative motion parameter to consider the influence of head motion occurring in the scanning period as a potential confounder, as previously described.^146^ Data were subsequently spatially normalized to the EPI unified segmentation template in the MNI coordinates derived from the SPM12 software (considering that EPI normalization is able to reduce variability across subjects)^147^ and resampled to 3 × 3 × 3 cubic voxels. Spatial smoothing with an isotropic Gaussian kernel (with a full width at half maximum of 8 mm) was applied. For network decomposition, the GIFT toolbox was used (https://trendscenter.org/software/gift/), and a spatially constrained multi-objective optimization independent component analysis with reference (MOO-ICAR)^148,149^ was used to obtain spatial maps for selected large-scale networks, including the DMN, the language network (LN) and the salience network (SN), from a recently published set of brain networks.^150^ Moreover, spatial references for the central executive subdomain of the triple network domain were considered the primary focus of targeted left DLPFC stimulation (Neuromark_fMRI_2.2, IC91, IC92, IC93; https://trendscenter.org/data/).^151^ Spatial maps are used as reference templates to calculate functional networks for each subject by maximizing independence in the context of the spatial constraint. These template maps include brain networks with a non-artefactual neuronal origin and classify the remaining data as noise. We utilized the recently published set of 12 spatial maps for our network selection, with consideration of the DMN, LN, SN and central executive subdomain of the triple network domain as the network of interest.^150^ MOO-ICAR pre-processing was used to estimate individual networks.^150^ The back-reconstruction step was used to consider the estimation of subject-specific networks and their related time courses based on the selected six networks (DMN, LN, SN and the executive subdomain of the triple network domain (IC91, IC92 and IC93)).^150-152^ For structural MRI analysis, a flexible factorial design was implemented in SPM12 to test the efficacy of the interventions, with consideration of the normalized and modulated rs-fMRI images of each assessed network (DMN, LN, SN and the central executive subdomain of the triple network domain (IC91, IC92 and IC93)) for the three groups of subjects (atDCS-Lang, ptDCS-Lang and atDCS-Cog) across two time points (T0, T1). Nuisance variables such as age, sex, disease stage (CDR plus NACC FTLD), TIV and FD were considered. A statistical threshold of *P* < 0.001 (uncorrected for multiple comparisons) was established, with a minimum cluster size of 100 voxels.

### Statistical analysis

Descriptive statistics at each time point are presented as the mean and standard deviation for continuous variables and as the amount (%) for categorical variables. Data from the two subjects who dropped out before the application of treatment in the primary outcome were not included in the analyses. Moreover, for the analysis, we employed mean or median imputation (depending on the variable distribution) to address the issue of missing observations at a specific time point. Due to the fact that the percentage of the missing values was small enough (a maximum of 5% of the overall distribution), we used a simple imputation method to preserve the marginal distributions of the variables while allowing for the use of the full sample.

To evaluate the efficacy of the interventions, we analysed data from three groups of subjects (atDCS-Lang, ptDCS-Lang and atDCS-Cog) across the three time points (T0, T1 and T2). Depending on the distribution of the variables (Gaussian, gamma, beta, Poisson, Tweedie or logistic), we employed GLMMs to assess score variations among the groups over time. Each model used a specific test score as the dependent variable, with time, group and the time × group interaction term as fixed effects.

To assess whether the treatment effect significantly differed between treated and untreated items, we separately analysed data from the three groups (atDCS-Lang, ptDCS-Lang and atDCS-Cog) and compared object naming performance in treated versus untreated conditions across the three time points (T0, T1 and T2). We employed GLMMs for the analysis, with three separate models being implemented (one model for each group). Object naming served as the dependent variable, whereas time, object lists (treated or untreated) and their interaction (time × treatment) were included as fixed effects.

Random effects for the subjects were added. *Post hoc* comparisons were adjusted using either the Tukey or Bonferroni methods (depending on the number of pairwise comparisons being simultaneously tested). Correlations between the variables were analysed by using Pearson’s r or Spearman’s rho coefficients based on their distribution in the overall avPPA sample. All of the statistical analyses were conducted using R software (version 4.3.2, R Core Team, 2013).

## Results

Descriptive statistics for the demographic variables are provided in Table 2. Statistical results for tests at each time point with relative cut-offs are presented in Supplementary Table 2. The GLMM analysis results are summarized in Supplementary Table 3. The three groups did not differ in terms of demographic, clinical, neuropsychological or language characteristics (Table 2; Supplementary Table 2). Similarly, a detailed qualitative error analysis focusing on naming subtests of the SAND also revealed no differences among the groups (Supplementary Table 4; Fisher’s exact test: *P* = 0.82), thus suggesting no noticeable divergence in the manner in which the naming error types are spread across the three groups (atDCS-Lang, ptDCS-Lang and atDCS-Cog). In particular, the prevalent type of error was anomia (36%), followed by phonological errors (25%, e.g. ‘gupo’ for gufo [owl]), semantic errors (26%, e.g. ‘delfino’ [dolphin] for foca [seal]), other errors (8%, e.g. ‘secchia’ for carriola [wheelbarrow]) and visual errors (5%, e.g. ‘borsa’ [handbag] for lucchetto [padlock]).

**Table 2:** Demographic and clinical characteristics collected at baseline (T0) in recruited avPPA patients

	All patients (*n* = 47)	atDCS-Lang (*n* = 16)	ptDCS-Lang (*n* = 16)	atDCS-Cog (*n* = 15)	*P*-value^a^
Age, years	68.6 (8.2)	67.1 (7.5)	69.4 (10.2)	69.4 (6.8)	0.665
Education, years	10.7 (4.0)	9.4 (4.0)	11.4 (3.8)	11.3 (4.1)	0.292
Gender (number of male/number of female)	21/26	6/10	7/9	8/7	0.672
Edinburgh handedness inventory (EHI)	86.3 (20.6)	86.9 (14.3)	82.3 (30.4)	90.0 (12.3)	0.689
CDR plus NACC FTLD	1.1 (0.6)	1.2 (0.6)	1.0 (0.5)	1.2 (0.6)	0.544
Cognitive reserve index-questionnaire (CRI-q)					
CRI-q total score	109.0 (18.0)	101.1 (17.3)	116.5 (17.8)	109.6 (16.2)	0.059
CRI-q, education score	102.2 (12.7)	98.1 (11.8)	104.9 (12.0)	103.8 (14.3)	0.302
CRI-q, working activity score	105.0 (18.0)	96.8 (13.1)	112.9 (20.0)	105.2 (18.0)	0.088
CRI-q, leisure time score	113.4 (22.2)	107.5 (21.0)	119.7 (22.6)	113.1 (22.7)	0.324

^a^ANOVA test for normally distributed variables; Kruskal–Wallis test for non-normally distributed variables; χ^2^ test for categorical variables. Raw scores mean are reported. Standard deviation between brackets. CDR plus NACC FTLD, global clinical dementia rating plus National Alzheimer’s Coordinating Center Frontotemporal Lobar Degeneration.

### Primary outcome

#### Additional gains from the combined treatment (atDCS-Lang) on performance in oral naming tasks

There were no significant differences observed in the baseline level of naming scores among the groups (*P* = 1 for atDCS-Lang versus ptDCS-Lang, *P* = 0.961 for atDCS-Lang versus atDCS-Cog and *P* = 0.919 for ptDCS-Lang versus atDCS-Cog (treated object naming task); *P* = 1 for atDCS-Lang versus ptDCS-Lang, *P* = 1 for atDCS-Lang versus atDCS-Cog and *P* = 0.982 for ptDCS-Lang versus atDCS-Cog (untreated object naming task)). All of the reported *P-*values have already been adjusted using the Bonferroni method.

With respect to additional gains from the combined treatment (atDCS-Lang) on performance in oral naming tasks, all of the groups demonstrated improvements in oral object naming from baseline (T0) to post-treatment (T1) (treated object naming task: β atDCS-Lang = +48.1, β ptDCS-Lang = +33.4 and β atDCS-Cog = +25.4; untreated object naming task: β atDCS-Lang = +31.6, β ptDCS-Lang = +19.7 and β atDCS-Cog = +29.8; *P* < 0.001 adjusted via the Tukey method for all of the T0-T1 pairwise comparisons); however, significant differences over time were observed among the three groups. Specifically, differences were noted in the treated object naming task from the IPNP (global time × group interaction: *P* = 0.008) and the untreated object naming task from the IPNP (global time × group interaction: *P* = 0.007).

**Figure 3:**
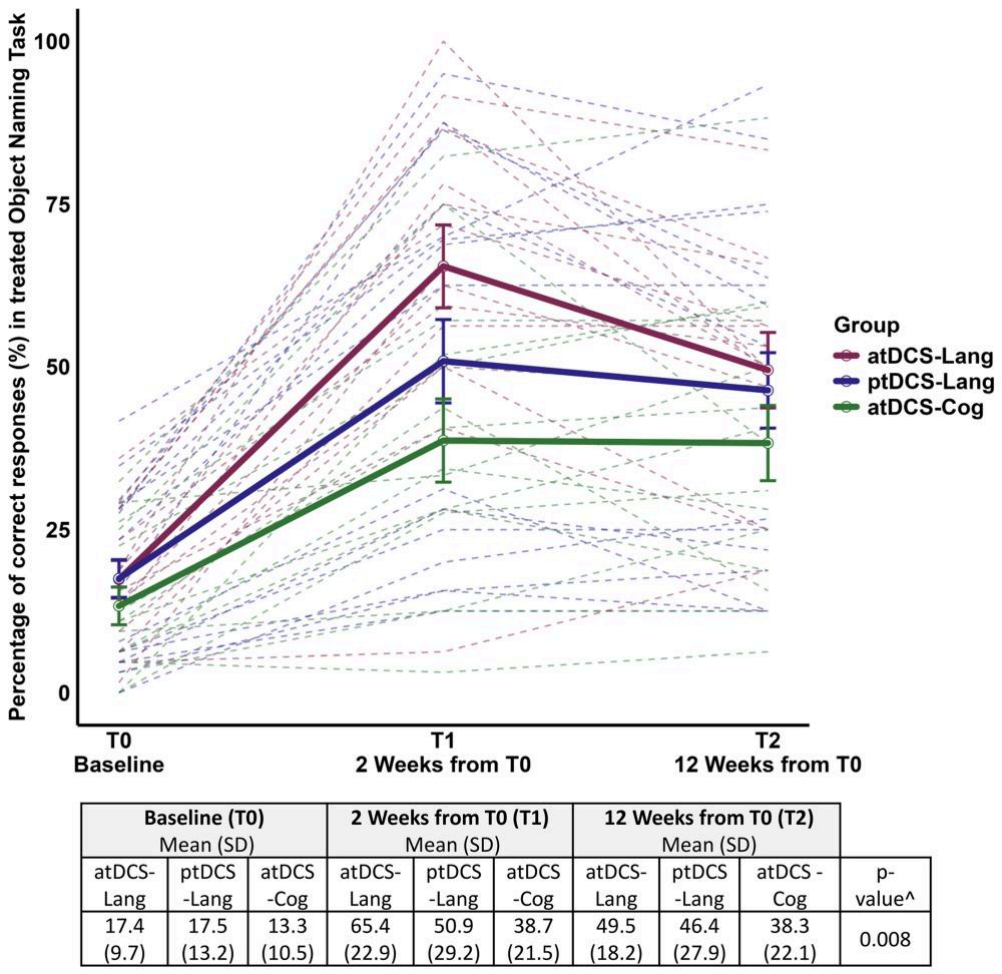
**Treated object naming task from IPNP (accuracy, %).** Effects of treatments on treated object naming task from IPNP. ^GLMMs showed a significant interaction between group and time (*P* = 0.008). All groups improved from baseline (T0) to post-treatment (T1) (*P* < 0.001, Tukey-adjusted). The atDCS-Lang group exhibited greater improvement compared to atDCS-Cog (*P* = 0.0007) and ptDCS-Lang (*P* = 0.029). Gains were maintained at follow-up (T2) without significant group differences. Dashed lines represent individual trajectories over time, with colours indicating the tDCS group. Each point corresponds to the outcome variable at a specific time point (*N* = 135; 15 observations per group and time point). Raw scores mean are reported. Standard deviation between brackets. atDCS-Cog, anodal transcranial direct current stimulation during unstructured cognitive stimulation; atDCS-Lang, anodal transcranial direct current stimulation combined with individualized language rehabilitation treatment; IPNP, International Picture Naming Project; ptDCS-Lang, placebo transcranial direct current stimulation during individualized language rehabilitation treatment; SD, standard deviation.

**Figure 4:**
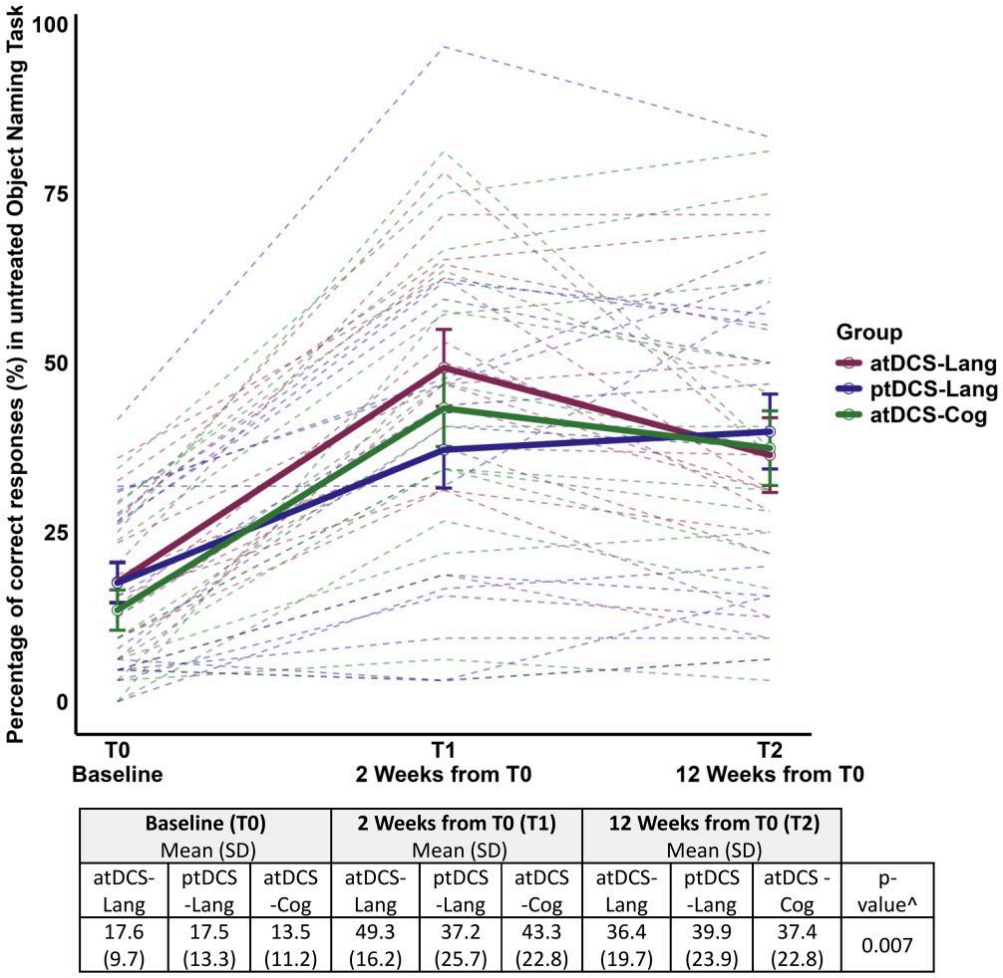
**Untreated object naming task from IPNP (accuracy, %).** Effects of treatments on untreated object naming task from IPNP. ^GLMMs revealed a significant group × time interaction (*P* = 0.007). All groups improved from T0 to T1 (*P* < 0.001, Tukey-adjusted), with atDCS-Lang (*P* = 0.025) and atDCS-Cog (*P* = 0.058) showing greater gains than ptDCS-Lang. Improvements were sustained at follow-up (T2) without significant group differences. Dashed lines represent individual trajectories over time, with colours indicating the tDCS group. Each point corresponds to the outcome variable at a specific time point (*N* = 135; 15 observations per group and time point). Raw scores mean are reported. Standard deviation between brackets. atDCS-Cog, anodal transcranial direct current stimulation during unstructured cognitive stimulation; atDCS-Lang, anodal transcranial direct current stimulation combined with individualized language rehabilitation treatment; IPNP, International picture naming project; ptDCS-Lang, placebo transcranial direct current stimulation during individualized language rehabilitation treatment; SD, standard deviation.

The atDCS-Lang group exhibited significantly greater improvement in the treated object naming task from T0 to T1 compared with both the atDCS-Cog (*P* = 0.0007, β = +22.7) and ptDCS-Lang groups (*P* = 0.029, β = +14.7) (see Fig. 3). Regarding the untreated object naming task, the atDCS-Lang group (*P* = 0.025, β = +12) and the atDCS-Cog group (*P* = 0.058, β = +10.2) exhibited significant and nearly significant improvements, respectively, from T0 to T1 compared with the ptDCS-Lang group, thus suggesting an effect of anodal tDCS on untreated object naming ability (see Fig. 4). There were no significant changes observed from T0 to T1 in any experimental group in the action naming task.

#### Long-term effects of the combined treatment (atDCS-Lang) on performance in oral naming tasks

All three groups (atDCS-Lang, ptDCS-Lang and atDCS-Cog) maintained gains from baseline (T0) and at 12 weeks from T0 (T2) for both the treated and untreated object naming tasks (treated object naming task: β atDCS-Lang = +32.1, β ptDCS-Lang = +28.9 and β atDCS-Cog = +25; untreated object naming task: β atDCS-Lang = +18.8, β ptDCS-Lang = +22.3 and β atDCS-Cog = +23.9; *P* < 0.001 adjusted via the Tukey method for all of the T0-T2 pairwise comparisons); moreover, no significant differences in long-term effects among the three groups were observed (see Figs. 3 and 4).

There were no significant changes observed from T0 to T2 in any experimental group in the action naming task.

#### Comparison of the effects of the treatments on the treated and untreated oral naming tasks

We compared the effects of the different treatment groups (atDCS-Lang, ptDCS-Lang and atDCS-Cog) on treated and untreated object naming. For the atDCS-Lang group, the time × object list (treated or untreated) interaction was significant, thereby indicating that the effect of treatment was significantly greater on the naming of treated objects than on the naming of untreated objects from T0 to T1 (*P* < 0.001, β = +16.4) and from T0 to T2 (*P* = 0.007, β = +10.7). Similarly, in the ptDCS-Lang group, the time × object list (treated or untreated) interaction was significant, thereby indicating that the effect of treatment was significantly greater on the naming of treated objects than on the naming of untreated objects from T0 to T1 (*P* = 0.001, β = +13.7) but not from T0 to T2. Conversely, no interaction effect was observed for the atDCS-Cog group.

### Secondary outcomes

#### Clinical, neuropsychological and language assessments

None of the clinical, neuropsychological or language assessment variables exhibited a significant group × time interaction (see Supplementary Table 3). However, significant time-dependent differences were observed across all of the groups, thus suggesting an improvement in all of the avPPA patient groups from T0 (baseline) to T1 (post-treatment) according to the Beck Depression Inventory (BDI)^115^ (Bonferroni-adjusted *P* = 0.011, β = −1.5); the Stroke and Aphasia Quality of Life Scale-39 (SAQOL-39) energy score^112^ (Bonferroni-adjusted *P* = 0.006, β = +0.5); and the Lincoln Questionnaire speech score^113^ (Bonferroni-adjusted *P* = 0.035, β = +0.8). Additionally, there was an improvement observed in all of the avPPA patient groups from T0 (baseline) to T2 (follow-up assessment) in the SAND-non-word repetition subtest score^106^ (Bonferroni-adjusted *P* = 0.008, β = +0.4).

#### BDNF and neurogranin plasma levels

**Figure 5:**
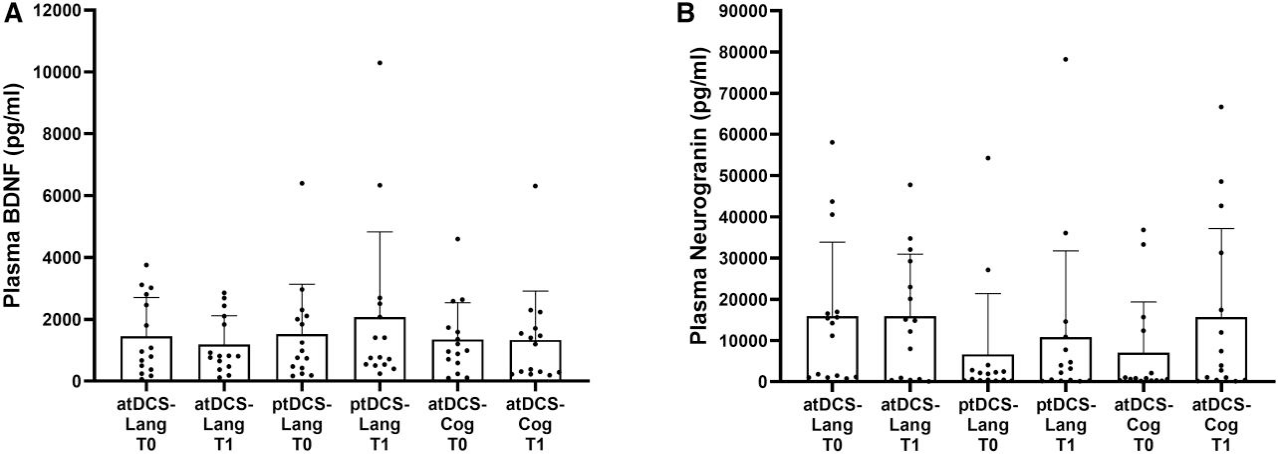
**Effects of treatments on BDNF and neurogranin levels.** (**A**) BDNF and (**B**) neurogranin levels at baseline (T0) and post-treatment (T1) in the three groups of treatment (atDCS-Lang, ptDCS-Lang and atDCS-Cog). GLMMs, global time × group interaction (*N* = 45), BDNF *P* = 0.645, Neurogranin *P* = 0.243. Each point represents plasma BDNF or Neurogranin concentration. atDCS-Cog, anodal transcranial direct current stimulation during computerized cognitive training; atDCS-Lang, anodal transcranial direct current stimulation combined with individualized language rehabilitation treatment; BDNF, brain-derived neurotrophic factor; pg/ml, picograms per millilitre; ptDCS-Lang, placebo transcranial direct current stimulation during individualized language rehabilitation treatment.

No significant differences in plasma BDNF or neurogranin levels over time were detected among the three groups of avPPA patients (atDCS-Lang, ptDCS-Lang and atDCS-Cog) (BDNF global time × group interaction: *P* = 0.645; neurogranin global time × group interaction: *P* = 0.243; see Fig. 5; Supplementary Table 5). The plasma BDNF and neurogranin levels at baseline (T0) and the variation from baseline to post-treatment (T1-T0) were not significantly correlated with performance in the treated and untreated object naming tasks (Supplementary Table 6). Moreover, the correlations between plasma BDNF and neurogranin levels (both T0 and T1-T0) and the secondary outcomes (clinical, neuropsychological and language assessments) were evaluated. Plasma BDNF variation (T1-T0) was positively correlated with the CRI-q working activity subscale (Spearman correlation, *r* = 0.304, *P* = 0.05; Supplementary Fig. 1, Panel A) and with the SAND-semantic association subtest (Spearman correlation, *r* = 0.298, *P* = 0.047; Supplementary Fig. 1, Panel B), thereby suggesting considerable BDNF variation from baseline to post-treatment in patients with greater cognitive reserve and preserved understanding of the meaning of words.

#### Structural MRI and functional connectivity fMRI analyses

No significant differences over time were observed in structural (VBM) or functional connectivity analyses (DMN, LN SN, or the executive subdomain of the triple network domain (IC91, IC92 and IC93)) among the three groups of avPPA patients (atDCS-Lang, ptDCS-Lang and atDCS-Cog) at the pre-established threshold (*P* < 0.001 uncorrected for multiple comparisons). For the ROI-based analysis (considering the left DLPFC), no significant differences in regional GMV were observed among the groups (main effect and interaction) for the pre-established threshold. When considering the partial correlation analysis, no significant relationship between naming improvements and left DLPFC volume differences (delta T1-T0) was demonstrated for the pre-established threshold.

## Discussion

PPA is a neurodegenerative condition involving progressive language decline, with increasing consequences on patients’ relationships, participation in communicative everyday activities and social networks.^5,153,154^ The agrammatic variant of primary progressive aphasia (avPPA) is characterized by slow, effortful and hesitant language production, including speech sound errors and poor grammatical comprehension.^7^

The main purpose of this study was to investigate whether the use of anodal tDCS (atDCS) applied to the DLPFC during individualized language training for 25 min a day at 5 days a week for 2 weeks would lead to significant naming improvements in patients with avPPA. Specifically, we hypothesized that atDCS plus individualized language training may improve the oral naming ability of treated and untreated objects compared with placebo tDCS plus individualized language therapy and atDCS combined with computerized cognitive training (atDCS-Cog). To address this question, we compared the effects of anodal or placebo tDCS plus individualized language training or atDCS combined with computerized cognitive training (atDCS-Cog) on patient performance in object naming tasks. Moreover, by directly comparing the three training groups, we aimed to observe additional gains induced by anodal tDCS. Another key objective of the present study was to assess whether the language improvements observed immediately after treatment (T1) would be sustained over time until 12 weeks from T0 (T2). Finally, we aimed to verify whether neuroimaging and blood marker changes were associated with improvements in language performance.

Overall, the results of our study revealed a significant improvement in oral naming task performance for treated object stimuli from baseline (T0) to post-treatment (T1), which was induced via atDCS coupled with individualized language rehabilitation treatment (atDCS-Lang), compared with the other protocols (ptDCS-Lang and atDCS-Cog).

One of the goals of treatment involves the generalization of effects to the oral naming of untreated objects. We observed a generalization effect to untrained object naming for all of the groups, although patients who received anodal tDCS (atDCS-Lang and atDCS-Cog groups) demonstrated greater improvements in the ability to name untreated stimuli from T0 to T1 compared with the group of patients who received placebo tDCS (ptDCS-Lang group), thus suggesting that anodal tDCS may be helpful in generalizing treatment effects to untreated stimuli. To our knowledge, this is the first study to compare the effects of language treatment combined with anodal or placebo tDCS with those of an intervention involving the combination of atDCS plus non-linguistic treatment on object naming in PPA. In this control condition, via atDCS and computerized cognitive training, it can be hypothesized that behavioural treatment does not directly affect naming ability, as it focuses on visuoperceptive and visuospatial cognitive functions. Therefore, the observed improvements in the naming of treated and untreated objects may be attributable to the induced effect of anodal tDCS.

Contrary to previous findings, the present study did not reveal a significant effect on action naming performance in individuals with avPPA. This outcome may be attributed to differences in the stimulation protocol employed. In our study, patients underwent daily tDCS combined with either object naming therapy or non-linguistic cognitive training. In contrast, recent studies that reported generalization effects in action naming applied tDCS to the left inferior frontal gyrus specifically during verb-focused treatment sessions.^81,155^

Conventional tDCS is a neuromodulation technique in which current intensities between 1 and 2 mA are applied through two electrodes (the anode and cathode) that are placed on the head. The primary effects of tDCS have been demonstrated to be derived from a shift in the membrane potential influenced by the electrical current direction; specifically, anodal tDCS increases neuronal excitability via depolarization of the resting potential, whereas cathodal tDCS hyperpolarises the resting potential, thereby suppressing neuronal excitability.^68,156^ The aftereffects induced by tDCS require synaptic plasticity, due to the fact that prolonged membrane polarization changes neuroplasticity through N-methyl-D-aspartic acid receptors, thereby leading to long-lasting aftereffects.^156,157^ The best manner of inducing brain modulation is to stimulate the cortical area of interest and simultaneously activate the network that supports the target function; this procedure can be achieved by combining tDCS (exogenous plasticity) with cognitive training (endogenous plasticity).^158-160^ Based on these reasons, the stimulation of the brain via tDCS during a focused specific cognitive task is expected to induce strong and more persistent effects over time. In our protocol, patients received tDCS during specific treatment that was not focused on verb rehabilitation; instead, treatment was focused on object naming abilities. Moreover, the lack of a generalization effect on action naming performance aligns with our previous studies on PPA, which did not observe any changes regarding action naming tasks.^74,75^ Several clinical observations have suggested that different cerebral areas are involved in the processing of nouns and verbs and that the naming of objects and actions involves multiple components, with a reliance on the functioning of various brain regions.^161,162^ Moreover, the generalization effect in PPA may depend on factors such as the progression of disease and the severity of the symptoms^76,77^; moreover, in a previous study, we reported that action naming improvement was related to grey matter density in the left middle temporal gyrus, thereby suggesting that intervention in early disease stages may be most successful.^74^

In addition, we tested the long-term beneficial effects of these rehabilitative interventions and reported that the improvement in the oral object naming task for both the treated and untreated stimuli was stable until 12 weeks from T0 (T2) in all three treatment groups. The synergy of these aspects (active tDCS, language and cognitive treatment and repeated sessions) likely played a major role in maintaining the effect. In particular, we hypothesize that several factors may contribute to the maintenance of the effects observed in the three patient groups: (i) the observation of a significant naming improvement with long-term maintenance in all of the experimental groups aligns with previous findings^72,75,76,78,81,86,163^ and could be explained by the fact that there was an intensive application (high frequency and many sessions) of the treatments in all of the groups (atDCS-Lang, ptDCS-Lang and atDCS-Cog)^80^; and (ii) the observed effects may be due to the specific individual language intervention that was applied, due to the fact that recent evidence suggests that impairment-based/restitutive approaches and compensatory strategies may be successful in PPA patients.^60^ Within this context, the literature highlights lexical retrieval treatment as the most commonly employed intervention for addressing progressive word-finding difficulties in individuals with PPA.^44,60^ This approach involves a hierarchical set of tasks aimed at promoting the strategic use of preserved semantic, orthographic and phonological knowledge to facilitate word retrieval and encourage self-cueing, ultimately enhancing naming abilities.^44,46,60^ Although the underlying mechanisms of anomia may vary, various approaches (including lexical retrieval training, phonological and orthographic treatment and semantic treatment) seem to achieve generalized benefits based on untreated stimuli across different variants of PPA.^48,138,140,164-177^ In particular, evidence suggests that phonological cueing treatment (similar to the language training applied in the present study) may further increase the possibility of generalizing the results to untrained items in patients with avPPA.^58,60^

Combinations of active (anodal) tDCS and treatment (such as lexical retrieval treatment or cognitive training) offer a safe and effective method for maintaining effects over time, thereby demonstrating potential as a tool for slowing the rate of decline in neurodegeneration.

With respect to the secondary outcomes, none of the other analysed variables exhibited significant differences over time among the three groups.

However, some secondary outcome variables, such as depressive symptomatology measured via the BDI scale and quality-of-life scales (including the SAQOL-39 energy subscale and scores obtained on the Lincoln Questionnaire), demonstrated a change in all of the avPPA scores. The latter result suggests that all of the intensive treatments proposed in the present study reduced depressive symptoms and improved quality of life and functional speech, although these improvements did not exhibit long-term maintenance.

Moreover, no significant differences over time were observed in either plasma BDNF or neurogranin levels among the three treatment groups, thereby suggesting that the proposed tDCS treatments combined with individualized language training or computerized cognitive training did not affect these specific markers of neuronal/synaptic plasticity. When considering the results of the MRI analyses, no significant clusters over time were observed in the three different groups for either the structural or functional analyses. If a structural effect mediated by neuromodulation and/or language training was potentially beyond the scope of the present study (particularly considering the relatively small time period of the intervention at 2 weeks), we would have expected an effect to be observed on functional connectivity. We were also unable to demonstrate a significant perturbation of brain connectivity induced by the implemented treatments; therefore, we hypothesize that more sophisticated approaches (such as time-varying functional connectivity and machine learning approaches) may demonstrate this effect, thus aligning with previous studies demonstrating a significant modulation of brain connectivity after tDCS in healthy and pathological brain specimens,^178-181^ which warrants the performance of future studies.

Recent studies have indicated that the main mechanism underlying the application of tDCS in PPA patients is primarily (or even exclusively) based on the modulation of cortical excitability via a combined approach that induces neuroplasticity by activating impaired linguistic functions in association with tDCS, which is able to persist after the end of stimulation.^39,75,77,79,80,182-184^ Thus, the combined application of specific cognitive training and tDCS is fundamental for inducing effective mechanisms of synaptic plasticity.^185-187^ Given the current scenario, the complementary use of tDCS with rehabilitation protocols may be further developed in the coming years as an important support strategy in the early stages of PPA.

The present findings concerning the combined application of tDCS and language therapy in PPA patients appear to align with the findings of a recent Cochrane review by Roheger *et al*., who demonstrated that the combination of anodal tDCS with language therapy may improve treated word retrieval beyond the effects of behavioural treatment alone.^188^ To investigate the effects of tDCS associated with speech treatment in PPA, further research is needed, including randomized controlled trials, which may contribute to the development of an optimized protocol for tDCS in the treatment of language disorders in neurodegenerative diseases, as was indicated by a recent meta-analysis. Specifically, Lomi *et al*.^189^ reported no statistically significant difference between active tDCS interventions and placebo interventions. Nevertheless, the authors acknowledged that available studies have obtained mixed results; moreover, due to the heterogeneity of the results and variations in rTMS and tDCS protocols, no definitive conclusions were obtained, thus indicating the need for further research. Moreover, the same analysis highlighted the fact that the combination of stimulation techniques with speech and language therapy can induce greater effects and that the parallel design outperformed the designs of crossover trials.^189^

The current study has several limitations that warrant discussion. First, the sample size was small, and an investigation of the neuropathological causes of the pathology at the individual level (such as the determination of the presence of Alzheimer’s disease biomarkers) was not available. Moreover, we could not definitively determine whether the naming improvements could translate into long-term enhancements in quality of life. Another potential limitation of the present study is the absence of control conditions in which tDCS was applied over different cortical areas. Moreover, the placement of the reference electrodes (either anodes or cathodes) on a cephalic region may induce reference-specific effects in addition to the effects of the active electrode.

Future studies based on larger patient samples with individual electric flow modelling (which may provide additional information regarding the relationship between field strength and intervention outcomes) and incorporating placebo and additional control conditions are warranted to determine the optimal parameters for combined treatment protocols. Further research is also needed to identify the patient profiles most likely to benefit from such interventions. Additionally, upcoming investigations should assess the functional changes in cortical reactivity and effective connectivity elicited by neuromodulation protocols.

In conclusion, the present study provides evidence that tDCS can facilitate oral naming performance in patients with avPPA. As indicated by the results, the short-term effects of anodal tDCS coupled with individualized language rehabilitation treatment were demonstrated from baseline to the immediate post-treatment assessment. More systematic research on both anodal tDCS and control groups (such as placebo tDCS and non-language training) is needed to characterize the long-term effects of atDCS on behavioural language measures and clarify its role in the neurorehabilitation of PPA. Currently, much research remains to be performed to investigate optimal modalities, brain targets and therapeutic approaches for specific language impairments in individuals with PPA.

These findings, although preliminary, support the efficacy of individualized language treatment coupled with tDCS for patients with avPPA.

## Supplementary Material

fcaf295_Supplementary_Data

## Data Availability

The data that support the findings of this study are available in Zenodo repository: https://zenodo.org/records/13642333.
