# Supplementary material for: A multi-modal approach for the treatment of non-fluent/agrammatic variant of Primary Progressive Aphasia
Source: Brain Commun. 2025 Sep 3;7(5):fcaf295. doi: 10.1093/braincomms/fcaf295 (PMC12405764; doi:10.1093/braincomms/fcaf295)
Supplement: fcaf295_Supplementary_Data [file fcaf295_supplementary_data.docx]

Supplementary Materials

**Supplementary Table 1.** **Computerised cognitive training exercises**

| **Main domain** | **Task and description** | **Task duration** |
| --- | --- | --- |
| **Visuospatial/ Visual-perceptual abilities** | *- Road route:* A road map is displayed on the screen and a ball runs a route. The participant has to pay attention to the ball and to reproduce the route; | 5 minutes |
|  | *- Puzzle:* Individual pieces of the puzzle are shown on the screen and the participant is requested to arrange them in order to compose the whole puzzle; | 5 minutes |
|  | *- Connections of points:* A series of circles with numbers or letters are randomly presented on the screen and the participant is invited to connect the circles in the correct sequence, following the numerical or alphabetical order; | 5 minutes |
|  | - *Umbrella*: An umbrella is displayed on the screen and raindrops start falling from above. The participant is requested to move the umbrella to the left or to the right to catch the drops; | 5 minutes |
|  | - *Complete the rotation sequence*: A sequence of rotated animals or arrow pictures is displayed on the screen. The patient is asked to select, from a set of options, the picture that correctly completes the rotation sequence; | 5 minutes |
|  | - *Avoid the obstacles*: A route surrounded by walls is displayed on the screen. The participant is requested to drag the ball from the starting area to the finishing area without touching the walls; | 5 minutes |
|  | - *Mirrored objects*: A chessboard is split in two sides by a line and animal pictures are displayed on one side. The participant is asked to place animal figures on the other side creating two symmetrical chessboard sides; | 5 minutes |
|  | - *Follow the path*: A path is displayed on the screen and the participant is requested to trace a trajectory as close as possible to the indicated one; | 5 minutes |
|  | - *UFO*: UFOs move around the screen and the participant is invited to hit them; | 5 minutes |
|  | - *Find the identical object*: The participant is requested to identify a target figure in a set of animals or arrow pictures presented on the screen. | 5 minutes |

**Supplementary Table 2. Descriptive statistics and relative cut-off for** **Clinical, Neuropsychological and Language Assessments of agrammatic variant of Primary Progressive Aphasia (avPPA) patients, grouped according to randomised treatment procedure, at baseline (T0), at posttreatment (T1) and at follow-up (T2).**

|  | **atDCS-Lang** | | | **ptDCS-Lang** | | | **atDCS-Cog** | | |  |
| --- | --- | --- | --- | --- | --- | --- | --- | --- | --- | --- |
|  | **T0**  Mean (SD) | **T1**  Mean (SD) | **T2**  Mean (SD) | **T0**  Mean (SD) | **T1**  Mean (SD) | **T2**  Mean (SD) | **T0**  Mean (SD) | **T1**  Mean (SD) | **T2**  Mean (SD) | Cut-off  (in the norm if) |
| **Clinical and Functional Assessment** | | | | | | | | | | |
| BDI | 8.7 (4.2) | 7.5 (4.6) | 7.9 (3.5) | 8.3 (4.3) | 6.9 (5.6) | 8.0 (5.2) | 8.7 (6.6) | 6.8 (5.1) | 7.1 (4.6) | <14 |
| FBI | 16.6 (12.3) | 15.6 (11.5) | 15.4 (13.1) | 11.7 (10.4) | 12.9 (13.1) | 13.6 (13.2) | 14.8 (8.3) | 13.4 (8.3) | 17.0 (10.7) | - |
| SAQOL-39 |  |  |  |  |  |  |  |  |  |  |
| - Total score | 3.9 (0.5) | 4.0 (0.6) | 4.0 (0.5) | 4.2 (0.4) | 4.2 (0.4) | 4.1 (0.5) | 4.1 (0.5) | 4.2 (0.4) | 4.2 (0.4) | - |
| - Physical score | 4.5 (0.4) | 4.3 (0.9) | 4.5 (0.7) | 4.7 (0.3) | 4.7 (0.5) | 4.5 (0.4) | 4.7 (0.4) | 4.7 (0.3) | 4.7 (0.2) | - |
| - Communication score | 3.1 (0.7) | 3.2 (0.9) | 3.3 (0.8) | 3.2 (0.7) | 3.3 (0.7) | 3.1 (0.8) | 3.5 (0.8) | 3.7 (0.8) | 3.5 (0.7) | - |
| - Psychosocial score | 3.5 (0.7) | 3.9 (0.5) | 3.7 (0.6) | 4.0 (0.8) | 4.1 (0.6) | 3.9 (0.7) | 3.8 (0.9) | 4.0 (0.8) | 4.0 (0.6) | - |
| - Energy score | 3.6 (1.1) | 4.2 (0.8) | 3.9 (1.1) | 4.2 (0.9) | 4.6 (0.7) | 4.1 (0.8) | 3.6 (1.3) | 4.0 (1.0) | 4.2 (1.1) | - |
| Lincoln Speech Questionnaire | |  |  |  |  |  |  |  |  |  |
| - Speech score | 8.9 (3.7) | 10.1 (2.6) | 9.6 (2.6) | 10.3 (3.7) | 11.4 (2.3) | 9.7 (3.1) | 8.7 (3.3) | 9.1 (3.9) | 9.2 (4.0) | - |
| - Understanding score | 3.5 (1.4) | 3.9 (1.2) | 3.7 (1.4) | 4.0 (1.3) | 3.9 (1.4) | 3.9 (1.1) | 3.5 (1.7) | 4.1 (1.1) | 3.6 (1.3) | - |
| ASRS | 1.4 (0.8) | 1.4 (0.8) | 1.3 (0.6) | 2.3 (1.5) | 2.1 (1.5) | 2.0 (1.5) | 1.5 (1.1) | 1.5 (1.1) | 1.4 (0.9) | - |
| **Neuropsychological Assessment** | | | | | | | | | | |
| **Memory** |  |  |  |  |  |  |  |  |  |  |
| Story Recall | 2.5 (2.3) | 3.2 (3.2) | 2.9 (2.9) | 3.6 (4.2) | 4.5 (4.7) | 5.2 (5.4) | 3.1 (3.1) | 3.7 (4.3) | 3.8 (5.0) | >7.5 |
| ROCF – Recall | 4.6 (2.5) | 4.2 (3.7) | 3.1 (2.5) | 4.8 (8.5) | 6.8 (8.7) | 6.2 (8.3) | 5.7 (5.1) | 6.5 (5.5) | 6.6 (7.2) | >9.46 |
| **Visuoconstructional abilities** |  |  |  |  |  |  |  |  |  |  |
| ROCF – Copy | 14.4 (10.4) | 13.4 (10.9) | 14.9 (10.5) | 13.7 (11.9) | 13.8 (12.5) | 12.6 (12.5) | 18.9 (10.4) | 18.5 (9.8) | 18.5 (11.4) | >28.87 |
| **Attentional and Executive functions** | | |  |  |  |  |  |  |  |  |
| TMT, Part A (sec) | 187.4 (95.5) | 200.3 (100.6) | 201.3 (104.1) | 196.3 (122.2) | 190.1 (118.5) | 206.4 (128.4) | 150.7 (92.5) | 142.8 (98.3) | 184.6 (110.9) | <127 |
| TMT, Par B (sec) | 543.6 (143.6) | 540.4 (157.6) | 536.0 (149.8) | 498.2 (175.3) | 520.8 (162.6) | 518.7 (163.3) | 503.1 (144.4) | 541.3 (125.9) | 517.3 (167.7) | <294 |
| **Language** |  |  |  |  |  |  |  |  |  |  |
| Phonemic Verbal fluency | 7.7 (6.8) | 8.1 (8.1) | 9.1 (8.2) | 8.9 (7.5) | 11.0 (10.4) | 8.9 (6.6) | 10.7 (10.8) | 10.5 (12.3) | 9.7 (11.8) | >16 |
| Semantic Verbal fluency | 10.1 (6.2) | 10.3 (6.8) | 10.3 (5.8) | 13.0 (9.9) | 13.0 (9.9) | 13.4 (11.1) | 12.9 (10.5) | 12.2 (10.4) | 10.8 (9.3) | >24 |
| Naming subtest from AAT | 86.1 (18.5) | 85.2 (24.4) | 84.7 (21.5) | 82.0 (24.9) | 82.3 (27.1) | 78.8 (30.2) | 80.9 (27.5) | 81.7 (29.4) | 75.9 (32.2) | >103 |
| IPNP - Action naming task (accuracy, %) | 47.7 (19.8) | 47.9 (21.5) | 49.7 (18.2) | 46.2 (31.0) | 49.0 (31.7) | 48.7 (32.2) | 48.2 (29.6) | 47.4 (29.2) | 45.5 (31.5) | - |
| IPNP – Treated Object naming task (accuracy, %) | 17.4 (9.7) | 65.4 (22.9) | 49.5 (18.2) | 17.5 (13.2) | 50.9 (29.2) | 46.4 (27.9) | 13.3 (10.5) | 38.7 (21.5) | 38.3 (22.1) | - |
| IPNP – Untreated Object naming task (accuracy, %) | 17.6 (9.7) | 49.3 (16.2) | 36.4 (19.7) | 17.5 (13.3) | 37.2 (25.7) | 39.9 (23.9) | 13.5 (11.2) | 43.3 (22.8) | 37.4 (22.8) | - |
| **Screening for Aphasia in NeuroDegeneration – SAND** | | | | | | | | | | |
| **Picture naming** |  |  |  |  |  |  |  |  |  |  |
| - Total score | 8.3 (4.2) | 8.3 (4.6) | 7.8 (3.5) | 8.6 (5.0) | 8.7 (5.4) | 8.5 (5.0) | 8.1 (5.1) | 8.1 (5.02) | 7.4 (4.5) | >9.969 |
| - Living score | 4.2 (2.3) | 4.3 (2.5) | 4.0 (1.9) | 4.3 (2.6) | 4.3 (2.8) | 4.3 (2.6) | 4.2 (2.7) | 4.4 (2.7) | 4.0 (2.5) | >3.829 |
| - Non-living score | 4.1 (2.1) | 4.0 (2.2) | 3.8 (2.0) | 4.3 (2.5) | 4.4 (2.7) | 4.2 (2.5) | 3.9 (2.6) | 3.7 (2.5) | 3.4 (2.2) | >5 |
| **Auditory sentences comprehension** | | |  |  |  |  |  |  |  |  |
| - Total score | 5.3 (1.9) | 5.2 (2.1) | 4.7 (2.2) | 5.3 (2.1) | 5.7 (2.1) | 4.9 (2.2) | 5.2 (2.2) | 5.5 (2.1) | 5.1 (2.8) | >6.157 |
| **Single word comprehension** |  |  |  |  |  |  |  |  |  |  |
| - Total score | 10.5 (2.1) | 10.0 (1.9) | 10.0 (1.5) | 10.1 (1.8) | 10.3 (1.8) | 9.5 (3.1) | 10.3 (1.8) | 9.9 (2.4) | 9.4 (2.4) | >10.258 |
| - Living score | 5.3 (1.1) | 5.1 (1.3) | 5.0 (1.1) | 5.3 (1.0) | 5.4 (1.1) | 5.1 (1.2) | 5.5 (0.7) | 5.3 (0.9) | 4.9 (1.4) | >5.048 |
| - Non-living score | 5.2 (1.3) | 4.9 (1.0) | 4.8 (1.3) | 4.8 (1.2) | 4.9 (1.3) | 4.9 (1.1) | 4.7 (1.2) | 4.5 (1.6) | 4.5 (1.5) | >4.876 |
| **Repetition** |  |  |  |  |  |  |  |  |  |  |
| - Total score | 6.7 (1.4) | 6.7 (2.0) | 7.8 (0.9) | 7.0 (2.1) | 7.5 (1.7) | 7.1 (2.1) | 6.2 (2.6) | 6.5 (2.4) | 6.1 (2.8) | >6.349 |
| - Words score | 5.6 (0.8) | 5.3 (1.2) | 6.0 (0.0) | 5.5 (0.8) | 5.7 (0.6) | 5.3 (1.2) | 5.1 (1.7) | 5.2 (1.5) | 4.8 (1.7) | >4.928 |
| - Non-words score | 1.1 (0.9) | 1.5 (1.1) | 1.8 (0.9) | 1.4 (1.6) | 1.8 (1.3) | 1.9 (1.4) | 1.1 (1.2) | 1.3 (1.3) | 1.3 (1.6) | >0.483 |
| **Sentence Repetition** |  |  |  |  |  |  |  |  |  |  |
| - Total score | 1.4 (1.0) | 1.3 (1.2) | 1.4 (1.0) | 1.7 (1.3) | 1.6 (1.3) | 1.8 (1.7) | 2.0 (1.6) | 1.9 (1.6) | 1.6 (1.2) | >2.455 |
| - Predictable sentence score | 0.9 (0.5) | 1.0 (0.7) | 1.0 (0.4) | 0.9 (0.6) | 1.1 (0.9) | 1.0 (0.8) | 1.1 (0.9) | 1.0 (0.8) | 1.0 (0.8) | >1.001 |
| - Unpredictable sentence score | 0.5 (0.6) | 0.3 (0.6) | 0.4 (0.7) | 0.8 (0.9) | 0.6 (0.5) | 0.8 (0.9) | 0.9 (0.8) | 0.9 (0.8) | 0.6 (0.5) | >0.784 |
| **Reading** |  |  |  |  |  |  |  |  |  |  |
| - Total score | 11.7 (4.6) | 12.1 (4.4) | 13.0 (2.9) | 12.5 (2.9) | 11.9 (3.8) | 12.2 (3.8) | 12.1 (4.8) | 11.7 (4.0) | 11.5 (5.3) | >13.489 |
| - Words score | 9.3 (3.6) | 9.4 (3.2) | 10.8 (1.4) | 9.9 (2.0) | 9.6 (2.8) | 9.9 (2.7) | 9.4 (3.6) | 9.6 (3.0) | 9.2 (3.9) | >10.106 |
| - Non-words score | 2.4 (1.4) | 2.7 (1.5) | 2.3 (1.6) | 2.6 (1.3) | 2.4 (1.2) | 2.3 (1.5) | 2.7 (1.3) | 2.1 (1.2) | 2.3 (1.7) | >2.228 |
| **Writing** |  |  |  |  |  |  |  |  |  |  |
| - Information Units score | 2.1 (1.3) | 2.3 (1.9) | 1.9 (1.1) | 2.7 (2.2) | 2.4 (2.1) | 2.5 (2.2) | 2.7 (2.1) | 2.8 (1.7) | 2.5 (1.6) | >2.132 |
| - Total number of words score | 6.8 (3.9) | 7.2 (5.2) | 8.1 (6.4) | 10.4 (13.1) | 9.6 (13.5) | 11.3 (13.9) | 13.8 (14.9) | 13.2 (8.7) | 18.4 (17.1) | >5.908 |
| - Number of nouns/total number of words score | 0.3 (0.1) | 0.3 (0.1) | 0.4 (0.2) | 0.4 (0.3) | 0.3 (0.3) | 0.4 (0.3) | 0.4 (0.3) | 0.3 (0.2) | 0.3 (0.3) | >0.15 |
| - Number of verbs/total number of words score | 0.2 (0.1) | 0.2 (0.1) | 0.2 (0.1) | 0.3 (0.1) | 0.2 (0.1) | 0.2 (0.2) | 0.2 (0.1) | 0.2 (0.1) | 0.3 (0.2) | >0.105 |
| - Number of correct syntactic structures/total number of syntactic structures score | 0.7 (0.4) | 0.5 (0.5) | 0.8 (0.4) | 0.5 (0.5) | 0.4 (0.5) | 0.4 (0.5) | 0.5 (0.5) | 0.6 (0.4) | 0.4 (0.4) | >0.75 |
| - Number of orthographic errors score | 4.8 (3.8) | 4.3 (4.3) | 3.2 (2.8) | 2.4 (3.2) | 2.4 (2.2) | 2.8 (4.1) | 3.2 (4.3) | 3.0 (4.5) | 5.5 (5.6) | <4.776 |
| - Number of lexico-semantic errors/number of words score | 0.0 (0.0) | 0.0 (0.1) | 0.0 (0.0) | 0.1 (0.1) | 0.1 (0.1) | 0.1 (0.1) | 0.1 (0.1) | 0.0 (0.1) | 0.1 (0.1) | <0.033 |
| **Semantic Association** |  |  |  |  |  |  |  |  |  |  |
| - Total score | 2.5 (0.9) | 2.3 (0.9) | 2.0 (1.0) | 2.7 (1.0) | 2.6 (1.4) | 2.5 (1.1) | 2.3 (1.2) | 2.3 (1.2) | 2.6 (1.1) | >1.166 |
| **Picture Description** |  |  |  |  |  |  |  |  |  |  |
| - Information Units score | 3.7 (2.1) | 3.5 (2.6) | 3.7 (2.2) | 4.1 (1.9) | 3.8 (2.4) | 4.4 (2.1) | 3.8 (1.9) | 3.7 (1.8) | 3.9 (2.7) | >3 |
| - Total number of words score | 73.9 (52.2) | 67.6 (49.4) | 76.2 (52.7) | 75.4 (36.3) | 71.2 (41.3) | 84.2 (32.5) | 74.7 (47.8) | 70.5 (45.2) | 80.1 (61.3) | >42.428 |
| - Number of nouns/total number of words score | 0.3 (0.2) | 0.3 (0.2) | 0.3 (0.1) | 0.2 (0.1) | 0.2 (0.1) | 0.3 (0.2) | 0.2 (0.1) | 0.3 (0.1) | 0.2 (0.1) | >0.198 |
| - Number of verbs/total number of words score | 0.1 (0.1) | 0.1 (0.1) | 0.2 (0.1) | 0.2 (0.0) | 0.2 (0.0) | 0.2 (0.1) | 0.1 (0.1) | 0.2 (0.1) | 0.2 (0.1) | >0.115 |
| - Number of sentences score | 5.0 (3.9) | 4.9 (4.7) | 5.2 (4.9) | 6.5 (4.2) | 5.9 (5.6) | 6.9 (5.4) | 4.8 (4.6) | 5.7 (3.9) | 6.3 (4.2) | >3.553 |
| - Number of subordinates/ number of sentences score | 0.2 (0.3) | 0.1 (0.1) | 0.2 (0.2) | 0.2 (0.3) | 0.2 (0.3) | 0.1 (0.2) | 0.0 (0.1) | 0.1 (0.2) | 0.1 (0.2) | >0 |
| - Number of repaired sequences/number of words score | 0.1 (0.1) | 0.1 (0.1) | 0.1 (0.1) | 0.1 (0.0) | 0.1 (0.1) | 0.1 (0.0) | 0.1 (0.0) | 0.1 (0.1) | 0.1 (0.1) | <0.121 |
| - Number of phonological errors/number of words score | 0.0 (0.0) | 0.0 (0.1) | 0.0 (0.0) | 0.0 (0.1) | 0.0 (0.1) | 0.0 (0.0) | 0.0 (0.0) | 0.0 (0.0) | 0.0 (0.1) | <0.0186 |
| - Number of lexico-semantic errors/number of words score | 0.1 (0.1) | 0.1 (0.1) | 0.1 (0.1) | 0.1 (0.0) | 0.1 (0.1) | 0.0 (0.1) | 0.1 (0.1) | 0.1 (0.1) | 0.1 (0.1) | <0.035 |

Raw scores mean are reported. Standard deviation between brackets. Cut-off scores according to Italian normative data are reported. sec= seconds. AAT = Aachener Aphasie Test; ASRS = Aphasia Severity Rating Scale; atDCS-Cog = anodal tDCS during computerised cognitive training; atDCS-Lang = anodal tDCS combined with an individualised language rehabilitation treatment; BDI= Beck Depression Inventory; FBI = Frontal Behavioral Inventory; IPNP = International Picture Naming Project; ptDCS-Lang = placebo tDCS during individualised language rehabilitation treatment; ROCF = Rey-Osterrieth Complex Figure; SAQOL-39 = Stroke and Aphasia Quality of Life Scale-39; tDCS = transcranial Direct Current Stimulation; T0 = Baseline Assessment; T1 = Posttreatment Assessment; T2 = 12 weeks from T0 Assessment; TMT = Trail Making Test.

**Supplementary Table 3. Results of GLMM models for Clinical, Neuropsychological and Language Assessments of agrammatic variant of Primary Progressive Aphasia (avPPA) patients.**

|  | ***p* Time** | ***p* Group** | ***p* Time x Group** |
| --- | --- | --- | --- |
| **Clinical and Functional Assessment** | | | |
| BDI | **0.013** | 0.942 | 0.865 |
| FBI | 0.341 | 0.279 | 0.377 |
| SAQOL-39 |  |  |  |
| - Total score | 0.228 | 0.204 | 0.375 |
| - Physical score | 0.243 | 0.566 | 0.371 |
| - Communication score | 0.447 | 0.140 | 0.688 |
| - Psychosocial score | 0.065 | 0.336 | 0.712 |
| - Energy score | **0.005** | 0.254 | 0.202 |
| Lincoln Speech Questionnaire |  |  |  |
| - Speech score | **0.017** | 0.308 | 0.145 |
| - Understanding score | 0.054 | 0.762 | 0.317 |
| ASRS | 0.052 | 0.993 | 0.241 |
| **Neuropsychological Assessment** | | | |
| **Memory** |  |  |  |
| Story Recall | 0.099 | 0.397 | 0.752 |
| ROCF – Recall | 0.530 | 0.640 | 0.543 |
| **Visuoconstructional abilities** |  |  |  |
| ROCF – Copy | 0.370 | 0.272 | 0.674 |
| **Attentional and Executive functions** |  |  |  |
| TMT, Part A (sec) | **0.011** | 0.523 | 0.208 |
| TMT, Par B (sec) | 0.083 | 0.999 | 0.478 |
| **Language** |  |  |  |
| Phonemic Verbal fluency | 0.808 | 0.957 | 0.537 |
| Semantic Verbal fluency | 0.181 | 0.570 | 0.514 |
| Naming subtest from AAT |  |  |  |
| IPNP – Action naming task (accuracy, %) | 0.982 | 0.928 | 0.721 |
| IPNP – Treated Object naming task (accuracy, %) | < 0.001 | 0.735 | 0.008 |
| IPNP – Untreated Object naming task (accuracy, %) | < 0.001 | 0.227 | 0.007 |
| **Screening for Aphasia in NeuroDegeneration – SAND** | | | |
| **Picture naming** |  |  |  |
| - Total score | 0.383 | 0.707 | 0.967 |
| - Living score | 0.647 | 0.909 | 0.892 |
| - Non-living score | 0.202 | 0.554 | 0.985 |
| **Auditory sentences comprehension** |  |  |  |
| - Total score | 0.092 | 0.905 | 0.883 |
| **Single word comprehension** |  |  |  |
| - Total score | 0.062 | 0.813 | 0.722 |
| - Living score | 0.565 | 0.579 | 0.527 |
| - Non-living score | 0.858 | 0.712 | 0.737 |
| **Repetition** |  |  |  |
| - Total score | 0.084 | 0.137 | 0.086 |
| - Words score | 0.223 | 0.685 | 0.683 |
| - Non-words score | **0.003** | 0.372 | 0.542 |
| **Sentence Repetition** |  |  |  |
| - Total score | 0.247 | 0.494 | 0.820 |
| - Predictable sentence score | 0.834 | 0.894 | 0.380 |
| - Unpredictable sentence score | 1 | 0.730 | 1 |
| **Reading** |  |  |  |
| - Total score | 0.289 | 0.933 | 0.570 |
| - Words score | 0.470 | 0.986 | 0.271 |
| - Non-words score | 0.276 | 0.927 | 0.410 |
| **Semantic Association** |  |  |  |
| - Total score | 0.481 | 0.479 | 0.145 |
| **Writing** |  |  |  |
| - Information Units score | 0.506 | 0.665 | 0.775 |
| - Total number of words score | 0.117 | 0.121 | 0.682 |
| - Number of nouns/total number of words score | 0.237 | 0.703 | 0.150 |
| - Number of verbs/total number of words score | 0.774 | 0.923 | 0.174 |
| - Number of correct syntactic structures/total number of syntactic structures score | 0.882 | 0.187 | 0.557 |
| - Number of orthographic errors score | 0.495 | 0.473 | 0.087 |
| - Number of lexico-semantic errors/number of words score | 0.990 | 0.843 | 0.953 |
| **Picture Description** |  |  |  |
| - Information Units score | 0.408 | 0.729 | 0.885 |
| - Total number of words score | 0.135 | 0.873 | 0.925 |
| - Number of nouns/total number of words score | 0.889 | 0.487 | 0.082 |
| - Number of verbs/total number of words score | 0.076 | 0.496 | 0.078 |
| - Number of sentences score | 0.538 | 0.476 | 0.586 |
| - Number of subordinates/ number of sentences score | 0.658 | 0.442 | 0.408 |
| - Number of repaired sequences/number of words score | 0.312 | **0.025** | 0.865 |
| - Number of phonological errors/number of words score | 0.828 | 0.624 | 0.287 |
| - Number of lexico-semantic errors/number of words score | 0.589 | 0.746 | 0.164 |

p-values of Generalized Linear Mixed Models (GLMMs) are reported. Significant fixed effects are highlighted. AAT= Aachener Aphasie Test; ASRS = Aphasia Severity Rating Scale; BDI = Beck Depression Inventory-II; FBI = Frontal Behavioral Inventory; IPNP = International Picture Naming Project; ROCF = Rey-Osterrieth Complex Figure; SAQOL-39 = Stroke and Aphasia Quality of Life Scale-39; Sec = seconds; TMT = Trail Making Test.

**Supplementary Table 4. Number and rate of error types in picture naming subtests of SAND (Screening for Aphasia in NeuroDegeneration) of agrammatic variant of Primary Progressive Aphasia (avPPA) patients, grouped according to randomised treatment procedure, at baseline (T0).**

| **Error types** | **All patients (n=47)** | **atDCS-Lang (n=16)** | **ptDCS-Lang (n=16)** | **atDCS-Cog (n=15)** | **p-value^** |
| --- | --- | --- | --- | --- | --- |
| Anomia | 91 (36%) | 27 (32%) | 30 (38%) | 34 (39%) | 0.82 |
| Semantic errors | 65 (26%) | 23 (27%) | 21 (27%) | 21 (24%) |  |
| Phonological errors | 62 (25%) | 22 (26%) | 15 (19%) | 25 (28%) |  |
| Articulatory errors/ Distorsions | 0 | 0 | 0 | 0 |  |
| Visual errors | 13 (5%) | 5 (6%) | 5 (6%) | 3 (3%) |  |
| Other types of errors | 20 (8%) | 7 (8%) | 8 (10%) | 5 (6%) |  |

^p-value from the Fisher’s Exact Test. Data are presented as follows: number of errors per type and their proportion relative to the overall errors in that group. atDCS-Cog = anodal tDCS during computerised cognitive training; atDCS-Lang = anodal tDCS combined with an individualised language rehabilitation treatment; ptDCS-Lang = placebo tDCS during individualised language rehabilitation treatment; tDCS = transcranial Direct Current Stimulation.

**Supplementary Table 5. Descriptive statistics for BDNF and Neurogranin levels of agrammatic variant of Primary Progressive Aphasia (avPPA) patients, grouped according to randomised treatment procedure, at baseline (T0) and at posttreatment (T1)**

|  | **Baseline (T0)**  Mean (SD) | | | | **Posttreatment (T1)**  Mean (SD) | | | |  |
| --- | --- | --- | --- | --- | --- | --- | --- | --- | --- |
|  | All patients  (n=45) | atDCS-Lang  (n=15) | ptDCS-Lang  (n=15) | atDCS- Cog  (n=15) | All patients  (n=45) | atDCS-Lang  (n=15) | ptDCS -Lang  (n=15) | atDCS-Cog  (n=15) | p-value^ |
| BDNF,  pg/ml  Neuro-granina, pg/ml | 1444.3 (1336.3)  9885.0  (15458,0) | 1456.0 (1257.0)  15980.5 (17888.3) | 1523.9 (1611.4)  6633.7 (14790.0) | 1352.8 (1191.5)  7040.7 (12347.0) | 1537.8 (1904.9)  14197.5  (19017.0) | 1190.4 (935.0)  15978.6  (14997.7) | 2081.2 (2750.6)  10887.6  (20857.7) | 1341.7 (1574.1)  15726.5  (21424.0) | 0.645  0.243 |

p-values of Generalized Linear Mixed Models (GLMMs) are reported. ^Interaction between group and time factor. Means and standard deviation (SD) between backets are reported. atDCS-Cog = anodal tDCS during computerised cognitive training; atDCS-Lang = anodal tDCS combined with an individualised language rehabilitation treatment; BDNF = Brain-derived neurotrophic factor; ptDCS-Lang = placebo tDCS during individualised language rehabilitation treatment; tDCS = transcranial Direct Current Stimulation.

**Supplementary Table 6.** Correlation between plasma BDNF and Neurogranin levels (T0 and T1-T0) and the performance in treated and untreated object naming tasks from IPNP in the overall agrammatic variant of Primary Progressive Aphasia (avPPA) sample (N = 45).

|  |  | **Plasma**  **BDNF**  **T0**  **(pg/ml)** | **Plasma Neurogranin**  **T0**  **(pg/ml)** | **Plasma**  **BDNF**  **(T1-T0)**  **(pg/ml)** | **Plasma Neurogranin**  **(T1-T0)**  **(pg/ml)** |
| --- | --- | --- | --- | --- | --- |
| Treated object naming task  T0 (accuracy, %) | Spearman r | -0.013 | -0.106 | -0.0422 | -0.146 |
|  | *p*-value | 0.930 | 0.487 | 0.783 | 0.338 |
| Untreated object naming task  T0 (accuracy, %) | Spearman r | -0.020 | -0.122 | -0.042 | -0.143 |
|  | *p*-value | 0.894 | 0.424 | 0.783 | 0.350 |
| Treated object naming task  T1-T0 (accuracy, %) | Spearman r | -0.100 | 0.224 | 0.035 | -0.061 |
|  | *p*-value | 0.512 | 0.139 | 0.821 | 0.692 |
| Untreated object naming task  T1-T0 (accuracy, %) | Spearman r | -0.071 | 0.017 | -0.116 | -0.001 |
|  | *p*-value | 0.645 | 0.910 | 0.448 | 0.995 |

Spearman correlation for non-normally distributed variables (Spearman r and p-value). BDNF = Brain-derived neurotrophic factor; IPNP = International Picture Naming Project; T0 = Baseline Assessment, T1 = Posttreatment Assessment.

**Supplementary Figure 1. Correlation between plasma BDNF variation (T1-T0) and Panel A CRI-q working activity and Panel B SAND-Semantic Association subtest.**


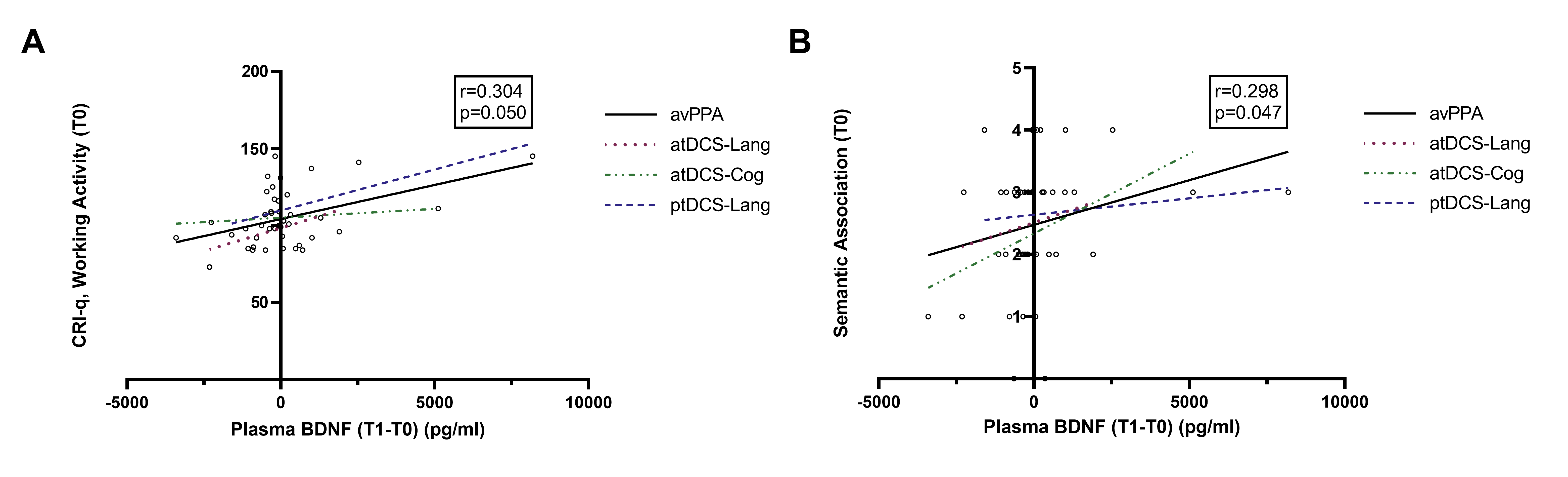


Spearman correlation (N = 45) (r and p-value). Each point represents plasma BDNF (T1-T0) and its corresponding CRI-q Working Activity (T0) score or semantic association (T0) score. atDCS-Cog = anodal transcranial Direct Current Stimulation during computerised cognitive training; atDCS-Lang = anodal transcranial Direct Current Stimulation combined with individualised language rehabilitation treatment; avPPA = agrammatic variant of Primary Progressive Aphasia; BDNF = Brain-derived neurotrophic factor; CRI-q = Cognitive Reserve Index-questionnaire; pg/ml = picograms per milliliter; ptDCS-Lang = placebo transcranial Direct Current Stimulation during individualised language rehabilitation treatment; SAND = Screening for Aphasia in NeuroDegeneration
